# Peroxisomal dysfunction interferes with odontogenesis and leads to developmentally delayed teeth and defects in distinct dental cells in *Pex11b*-deficient mice

**DOI:** 10.1371/journal.pone.0313445

**Published:** 2024-12-09

**Authors:** Claudia Colasante, Julia Jednakowski, Klaus-Peter Valerius, Xiaoling Li, Eveline Baumgart-Vogt

**Affiliations:** 1 Institute of Anatomy and Cell Biology, Justus-Liebig-University, Giessen, Germany; 2 The Laboratory of Signal Transduction, National Institute of Environmental Health Sciences, Research Triangle Park, Durham, North Carolina, United States of America; University of Sao Paulo: Universidade de Sao Paulo, BRAZIL

## Abstract

Human peroxisomal biogenesis disorders of the Zellweger syndrome spectrum affect skeletal development and induce tooth malformations. Whereas several peroxisomal knockout mouse studies elucidated the pathogenesis of skeletal defects, little information is available on how dental pathologies arise in peroxisomal biogenesis disorder patients. To understand the impact of severe peroxisomal dysfunction on early odontogenesis, here we performed morphometric studies on developing molars of new-born *Pex11b* knockout mice. Immunofluorescence analysis revealed reduced peroxisome number and mistargeting of the peroxisomal matrix enzyme catalase to the cytoplasm in several dental cell types of the *Pex11b* knockout animals. We also observed secondary mitochondrial alterations, comprising decreased staining of mitochondrial superoxide dismutase and of complex IV in cells of the developing molar. The peroxisomal defect caused by the PEX11b knockout also decreased the staining of cytokeratin intermediate filaments and of the secretory proteins amelogenin, osteopontin and osteocalcin. Interestingly, the staining of the gap junction protein connexin 43, an important modulator of tissue development, was also decreased, possibly causing the observed cellular disarrangement within the inner enamel epithelium and the odontoblast palisade. Taken together, our results show that the severe phenotype associated with the PEX11b knockout results in a reduction of the number of peroxisomes in dental cells and causes a delay odontogenesis. This adds a new component to the already described symptomatic spectrum induced by severe peroxisomal defects.

## Introduction

The importance of peroxisomes for the homeostasis of mammalian tissues is emphasized when these organelles become dysfunctional. Peroxisomal dysfunction arises by genetic mutations, metabolic dysregulation, drug treatment or environmental stress. In contrast to mitochondrial β-oxidation, which mainly degrades nutritional fatty acids to generate ATP, peroxisomal α- and β-oxidation protect the cell against the accumulation of insoluble, bioactive, proinflammatory or toxic lipid derivatives [1]. Peroxisomes also degrade reactive oxidative species (ROS) either by enzymatic reactions (catalase, peroxiredoxins 1 and 5) [2,3] or by synthesizing molecules to trap them (plasmalogens and polyunsaturated fatty acids) [1,4]. Mutations in genes coding for proteins of peroxisomal biogenesis and metabolism result therefore in severe genetic diseases named “peroxisomal disorders” [2,5–7]. The first described and most severe peroxisomal biogenesis disorder (PBD) is the Zellweger syndrome [8], followed in severity by the neonatal adrenoleukodystrophy and, the mildest form, the infantile Refsum´s disease. All are classified as Zellweger syndrome spectrum disorders, a set of genetically heterogeneous human conditions caused by mutations in 13 different peroxin genes (*PEX*) [9–13]. At birth, children with severe Zellweger syndrome exhibit generalized hypotonia, neonatal seizures and craniofacial dysmorphism comprising high forehead, low nasal bridge and hypertelorism. Frequently these children die within the first year of life due to organ malformations and metabolic dysfunction [9,14]. Ossification defects and malformations of the dental apparatus like dysgnathia, delayed eruption and malposition of the teeth as well as amelogenesis imperfecta were also diagnosed in juvenile patients with milder forms of Zellweger syndrome or Refsum´s disease [15–19]. Nevertheless, next to no studies were undertaken to clarify how peroxisomal diseases affect odontogenesis and development of the alveolar bone.

To investigate how peroxisomal dysfunction affects human health, several peroxin general knockout mouse models were established [20–24] including two mouse models affecting peroxisomal proliferation (PEX11a, PEX11b) [20,21]. Proteins of the PEX11 family (PEX11a, b and g) are central regulators of peroxisome proliferation and abundance through their interaction with proteins of the machineries for organellar fission, elongation and autophagy [25–27]. Interestingly, *Pex11a* knockout mice are viable until adulthood while the *Pex11b* knockout animals display similar symptoms as Zellweger syndrome patients [20,21]. The severe peroxisomal defect caused by the knockout of *Pex11b* induces a lethal phenotype in new-born mice. Symptoms including neuronal migration defects, elevated neuronal apoptosis, growth retardation and general hypotonia lead to premature death within the first day post-partum substantiating the importance of PEX11b for cellular health [20,21,28]. Importantly, the *Pex11b* knockout mice also exhibit ossification defects of the calvaria, a common disturbance of bone tissue development [20,21]. In this respect it is of interest, that an earlier study from our group demonstrated that *Pex11b* mRNA is highly expressed in all bones whether derived by chondral ossification (cartilage and osteoblasts of vertebrae, ribs and femur) or by desmal ossification (osteoblasts of the calvaria) [29]. Interestingly from the aspect of the development of teeth, the *Pex11b* mRNA was particularly abundant in the osteoblasts of the mandible (alveolar bone) [29]. We were therefore intrigued to investigate whether the peroxisomal defect caused by the knockout of *Pex11b* had also an impact on odontogenesis and alveolar bone development.

Odontogenesis is a process that has been comprehensibly characterized on the morphological level. However, its molecular and metabolic regulatory mechanisms are not yet fully understood. Briefly, during embryonic development, tooth buds are built from the interaction of the oral epithelium with the nearby mesenchymal tissue [30]. Odontoblasts differentiate from mesenchymal cells and build predentin, which mineralizes into dentin [31]. In contrast to odontoblasts, ameloblasts differentiate from epithelial cells and secrete enamel [32]. Murine odontogenesis follows the same order as in humans, therefore mouse models are suitable tools to investigate tooth development in mammals [33].

We previously showed that in new-born and adult mice peroxisomes can be detected in all cell types of dental tissues comprising ameloblasts, cells of the stratum intermedium, odontoblasts and pulp cells [34]. In the current study we analyzed the impact of a severe peroxisomal defect on odontogenesis using the *Pex11b* knockout mouse as an experimental model for the Zellweger syndrome.

## Materials and methods

### Ethics declaration

Procedures involving animals sacrificed and embedded at the JLU-Giessen (Justus-Liebig-Universität Giessen, Germany) were approved by and performed in accordance with the German Governmental Animal Care Committee (JLU internal classification: JLU No.: 616_M, Project ID: 1016 Peroxisomen). All protocols used during previous (from 2002) tissue collection to generate the paraffin blocks obtained from Johns Hopkins University (Baltimore, MD, USA) (for reference see [20,21]) were approved by the institutional animal ethics committee of the Johns Hopkins School of Medicine, and the method of anesthesia (ether) was appropriate according to the AVMA guidelines in effect at the time and performed following laboratory health and safety regulations (AVMA guidelines from 2000 and 2007).

### Animals and tissue isolation and embedding

The tissue blocks from 1 new-born wild type (WT), 1 heterozygote (HET) and 4 knockout (KO) mice (P0.5) of the B6.129-Pex11b^tm1Sjg^ line (knockout of *Pex11b* gene—MGI:1338882) were genotyped, prepared and embedded in paraffin by Eveline Baumgart(-Vogt) and Xiaoling Li in 2002 at the Johns Hopkins University (Baltimore, MD, USA) (For reference see [20,21]) and the blocks were transferred to professor Baumgart-Vogt´s laboratory at the Justus Liebig University Giessen (JLU, Hessen, Germany). The genotype of these animals was rechecked, by isolating DNA from the paraffine embedded tissue sections using the QIAamp DNA FFPE Advanced Kit according to manufacturer´s protocol. The corresponding DNA fragments for the genotyping (See [21] and S1A and S1B Fig) were amplified using multiplex PCR and the primers P8 (5 -GTCTAGGACAGGCTTCTGCTGTTC-3) P9 (5 -GTTTCCCCATCTTTCCCTTGAG-3) and PNeo (5 -ATATTGCTGAAGAGCTTGGCGGC-3) as previously described [21] (S1D Fig).

Additionally, after transfer and establishment of the B6.129-Pex11b^tm1Sjg^ mouse line [21] at the JLU animal facility, 3 WT and 3 HET mice (P0.5) were bred under standard conditions according to the Animal Care Commission (JLU-number 616_M) and genotyped using PCR and the 3 primer mentioned above (S1C Fig). These mice were perfusion-fixed and embedded at the JLU. At both laboratories perfusion-fixation and paraffin-embedding were performed using similar materials and technical procedures to guarantee comparability of results. Briefly, the mice were obtained from the according animal facilities on the day of birth (P0.5), anaesthetized with 2% isoflurane (or, in 2002, ether at the Johns Hopkins University [20,21]) via a gas diffusor and euthanized by a cut through the cervical spinal cord at the neck region. Thereafter, the thorax of the pups was opened using small scissors and perfusion-fixation was performed through the left ventricle of the heart using a microflow needle 0.5 x 20 mm (25G). For this purpose, a butterfly needle connected to a PBS-filled tube was coupled to 1 ml syringe containing 4% formaldehyde. After insertion of the needle into the left ventricle, the right atrium was opened, and the blood flushed away with PBS followed by slow and cautious perfusion with appropriate low pressure with 4% formaldehyde in the syringe (ca. 5 min). Thereafter the whole animals were median sagittalized and both halves were embedded (medial plane facing down) into paraffine (Paraplast+). In accordance to previously performed morphometric studies by our group a total of 4 wildtype, 4 heterozygous and 4 knockout mice were investigated to obtain statistically evaluable data. All experiments with laboratory mice were approved by the Commissions of Animal Care of the Justus Liebig University and Johns Hopkins University.

### Hematoxilin-Eosin (HE) staining

To inspect dental morphology at P0.5, 5 μm sections were cut using a microtome and stained with hematoxilin-eosin (HE). The sections were deparaffinized over night at 60°C. Then they were dipped 3 times for 3 min into Xylene for complete deparaffination. For rehydration the slides were treated with descending concentrations of ethanol (2x 99%, 96%, 90%, 80%, 70%, 50%, 2x aqua dest.) for 3 min each. The nuclei were stained using hematoxylin for 5 min. Afterwards the slides were washed under running water for 10 min. The cytoplasm was stained with eosin for 3 minutes followed by washing in running water. Prior to embedding slides were incubated with ascending concentrations of ethanol: 50%, 70%, and 80% ethanol (1 min each), 96% ethanol (3 min), and 99% ethanol (2 times 3 min). Finally, the slides were immersed in Xylene, allowed to dry and mounted in DEPEX.

### Immunofluorescence analysis

Prior to the final staining used for image acquisition and morphometric analysis each antibody was tested in a serial dilution using the tissue sections from the 3 genotypes and the immunofluorescence protocol described below. In this way the best concentration to use for each individual primary and secondary antibody combination was determined to avoid oversaturation or loss of signal during image acquisition.

For the immunofluorescence analysis 1–2 μm sections were cut using a microtome, layered on microscopy slides and deparaffinized overnight at 60°C followed by 3 times 5 min incubation in xylol. Tissue sections were rehydrated using descending concentrations of ethanol (2 x 99%, 96%, 80%, 70%, 50% and 2 x aqua dest.) for 3 min each. Sections were pre-treated with 0.01% trypsin in PBS for 14 min followed by 3 times 5 min washes with PBS. Then the slides were incubated for 3 times 5 min in 10 mM citrate buffer pH-6, in a microwave (900 W). After 30 min cooling, the slides were washed in PBS, 3 times for 5 min. To block non-specific binding sites the sections were dried, encircled with a PAP-pen and treated with PBS-Albumin (4%) (PBSA) containing 0.05% Tween 20-solution for 2 h in a moist chamber at room temperature (RT). Slides were then incubated with primary antibodies (see S1 Table in S1 File) diluted in 4% PBSA, 0.05% Tween-20 in PBS overnight at RT followed by a 2 h incubation with secondary antibodies (see S2 Table in S1 File) diluted in 1% PBSA and 0.05% Tween-20. Afterwards the slides were washed in PBS-buffer 3 times for 5 min. To stain the nuclei, slides were incubated with DAPI (Sigma-Aldrich) in PBS-buffer (1 μg/ml) for 10 min and then washed with PBS, 3x for 5 min. We used DAPI-staining to analyze the number of nuclei in mouse teeth. Microscopy and image acquisition were performed using a Leica TCS-SP2-laserscan microscope (all images included in the paper and S4, S5 and S13 Figs) or the STELLARIS Confocal Microscope Platform from Leica (All detailed and overview images depicted in S6–S12, S14 and S15 Figs).

### Morphometry and statistical analysis

To quantitatively analyze the acquired images, the program Image J (Version 1.52a) [35] was used to count the number of nuclei and peroxisomes in mandibular and maxillary first molars (S2 Fig). All steps described in this section as well as image recording were consistently performed by one single person to ensure the reproducibility of the results. After setting the correct scale (μm) for the image to be analyzed, the tissue region of interest (ROI) was determined (μm^2^) using the selection and measurement tools and organelles were counted within the ROI. The number of the nuclei (nuclei/μm^2^) was determined using the “Multi-point” tool of image J for manual counting after selecting and measuring the ROI (S2A Fig).

The number of peroxisomes was analyzed using the automatic “particle counting” function of Image J (S2B Fig). To distinguish the organelles from the background (Step 1., S2B Fig), the images were converted into 8-bit images (Step 2. S2B Fig) and then the threshold value was set (Step 3. S2B Fig). For each analyzed marker the threshold level was individually set to a value that allows detection of the organelles in all 3 genotypes. Individual peroxisomes that appeared to be fused were separated by converting the image to binary and applying watershed (Step 4. S2B Fig). To correctly allocate peroxisomes, the converted images were individually compared to the original immunofluorescence images. After setting the scale (μm) and selecting the ROI (Step 5. S2B Fig) all particles with intensities above the threshold value and with a size range of 0.01–1 μm^2^ were automatically counted by image J (Step 6. S2B Fig).

The program GraphPad Prism (GraphPad Prism 10, Version 10.1.1 (270), November 21, 2023) and the one-way ANOVA with Tukey-post-hoc-test (α = 0.05) were used to calculate the medians and the standard deviations and to define the statistical significance shown in the charts. In total, we analyzed 4 wildtype, 4 heterozygous and 4 knockout animals of the *Pex11b* mouse line. The specific numbers of individually analyzed images per genotype is indicated in the corresponding figure legends.

### RNA isolation from FFPE tissue sections and RT-qPCR analysis

The entire first molar from FFPE embedded wildtype, heterozygous and knockout mice (S3 Fig, Step 1.) was dissected from 10 μm thick tissue sections using a scalpel and razor blade to remove superfluous tissue (S3 Fig, Step 2.). The molars were picked up using a needle and the RNA was isolated from the tissue using the RNeasy^®^ FFPE Kit from Qiagen according to manufacturer’s protocol. Briefly, molars were deparaffinized using 160 μl of the provided deparaffinization solution and incubated for 3 min at 56°C. After Proteinase K and DNAse treatment, the lysate was loaded on a RNeasy MinElute spin column and centrifuged at 13000 x g. Following two consecutive washing steps, the RNA was eluted using 14 μl of RNase-free water.

First-strand cDNA was synthesized from 50 ng total RNA using oligo (dT) 12–18 primers and the High-Capacity cDNA Reverse Transcription Kit (Applied Biosystems—Thermo Fisher, Germany) with RNase Inhibitor (Invitrogen, Germany) according to the manufacturer’s protocol. Reverse transcription PCR was performed in the Bio-Rad T100 Thermal Cycler (Bio-Rad Laboratories, München, Germany) using the following parameters: 25°C for 10min, 37°C for 120min, 85°C for 5min and cooling to 4°C.

SSOAdvanced Universal SYBR^®^Green Supermix (Bio-rad) mixed 1:1 with the template cDNA, the forward and reverse primers and water. All samples were run in duplicates. The qPCR reaction was performed using the CFX Duet (Bio-Rad Laboratories, München, Germany) using the following 2-step amplification protocol: 3 min at 95°C (denaturation), 45 cycles of 10 s at 95°C (denaturation), 30 s at 60°C (annealing+extension). Intron spanning primer pairs were verified for specificity by melting curve analysis. For *Pex11b* we used forward primer GCCCAGTATGCCTGTTCCC, reverse primer CTCCAGTTGTCGAATCTGTTTCT. For *b-actin* we used forward primer GCTCCTCCTGAGCGCAAG, reverse primer CATCTGCTGGAAGGTGGACA. Calculations of the relative gene expression were done by the 2^-ΔΔct^ method (Pfaffl 2001) using *b-actin* as internal standard.

## Results

### Histological staining hints to differences in the developmental status of the molars of the PEX11b molars

**Fig 1:**
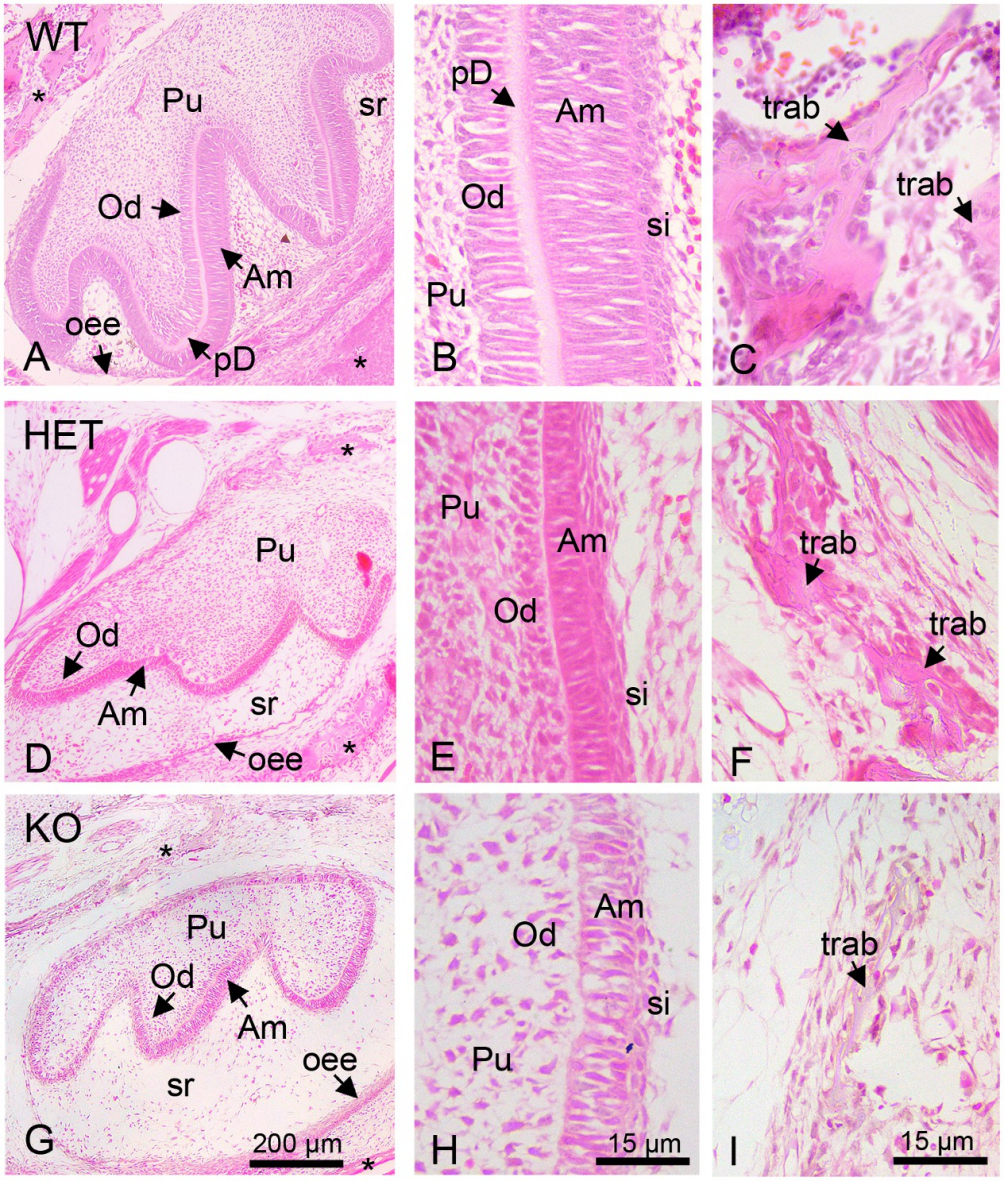
HE stainings of P0.5 mice first molars. (A-I): HE-stainings of whole first molars (A, D and G), higher magnification of the ameloblast/odontoblast mineralization front of the first molars (B, E and H) and of the alveolar bone (C, F and I) of wildtype (WT) (A-C), heterozygous (HET) (D-F) and knockout mice (KO) (G-I). The HE staining highlights the tissue structures and dental cell types that can be discerned during the bell stage of odontogenesis: Outer enamel epithelium (oee), stellate reticulum (sr), stratum intermedium (si), ameloblasts (Am), odontoblasts (Od), dental pulp (P), alveolar bone (*), predentin (pD) and bone trabeculae (trab).

At P0.5 murine first molars are in the bell stage of dental development. At this stage, the odontoblast and ameloblast layers can be well differentiated and dentin and enamel are starting to form. The future shape of the first molars with the three typical cusps and differentiated cell layers can be observed in the bell stage of all mouse pups (P0.5) using HE-staining (Fig 1A, 1D and 1G). The enamel organ consists of the outer enamel epithelium (synonym: external enamel epithelium), the stellate reticulum, the stratum intermedium and the inner enamel epithelium (synonym: internal enamel epithelium) differentiating into the ameloblast layer. Cranial ectomesenchymal cells give rise to the odontoblasts, which will secrete the dentin, and to the pulp.

Our microscopic analysis showed that the first molars of the wildtype mice displayed the typical cell layers expected during odontogenesis in newborn mice. As depicted in Fig 1, the ameloblast and odontoblast layers were already well developed and the predentin could be observed between the two layers (Fig 1A and 1B). Also, several alveolar bone trabeculae were observed surrounding the first molar (Fig 1A and 1C).

In comparison, in the first molars of heterozygous and knockout mice, the ameloblast and odontoblast layers appeared to be more disorganized (Fig 1A–1I). This was particularly evident in those areas in which the dental cell layers were sectioned in longitudinal plane as observed in the magnified images shown in Fig 1B, 1E and 1H. The odontoblast layer of the heterozygous molars and, more evidently the one of the knockout molars did not display the typical palisade-like organization that is common in the wild type molars of this stage (Fig 1B, 1E and 1H). Moreover, inspection of the still developing alveolar bone showed thinner bone trabeculae in heterozygous and knockout animals (Fig 1C, 1F and 1I).

**Fig 2:**
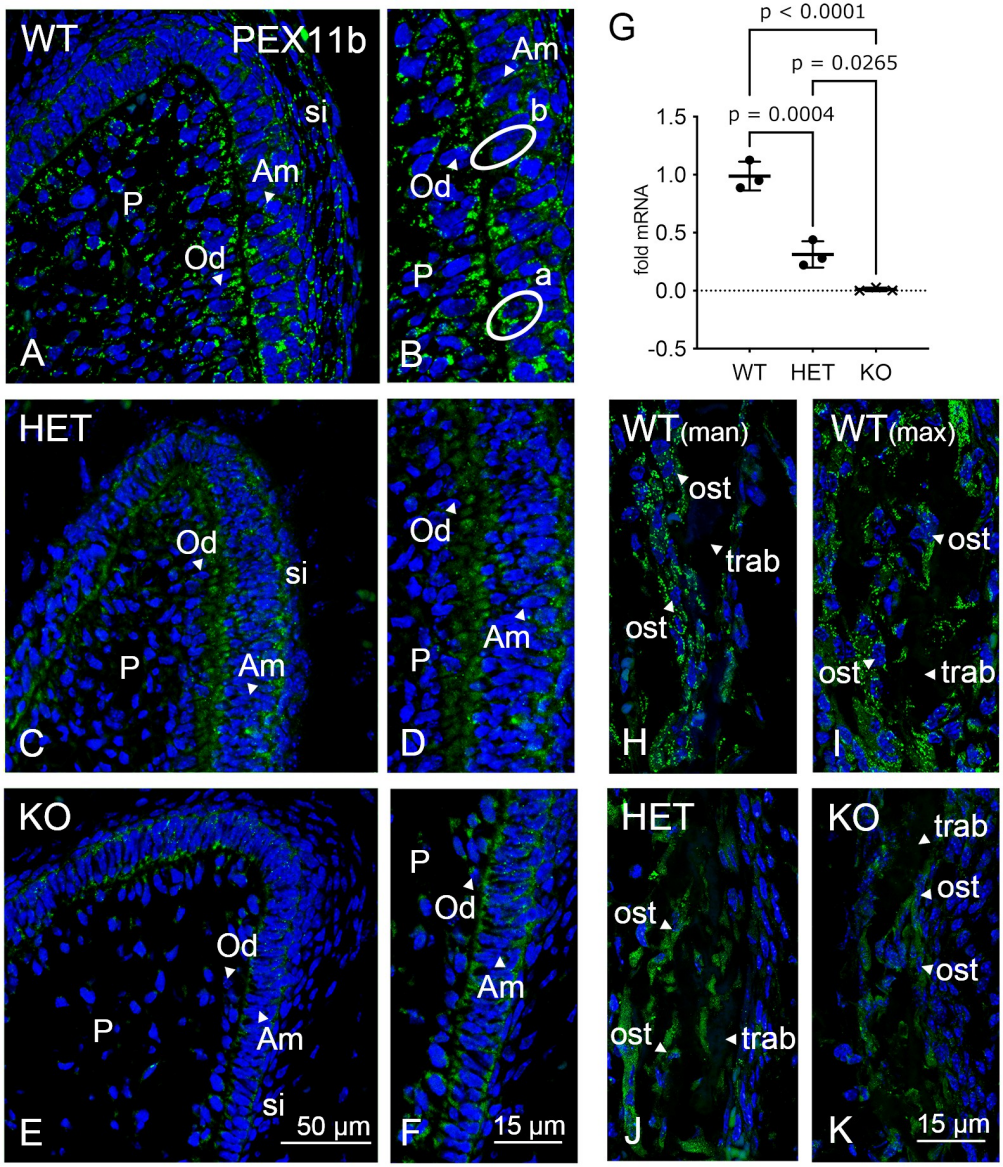
Immunofluorescence analysis of PEX11b in dental tissue. (A-F): Molars of wildtype, *Pex11b*-heterozygous and -knockout mice were stained with an antibody directed against PEX11b. Bars shown in figures (E) (overview images of a dental cusp) and (F) (detailed images of odontoblast and ameloblast layers) represent the magnification for the corresponding image columns of all three genotypes. In all images (A-F) DAPI was used to counterstain the nuclei (Blue). (G) mRNA expression of the *Pex11b* gene investigated by qPCR analysis. (H-K) Alveolar bone of wildtype (maxilla and mandible) and of the maxilla of *Pex11b*-heterozygous and -knockout mice were stained with an antibody directed against PEX11b. Bars shown in figures (K) represent the magnification all alveolar bone images. In all images (H-K) DAPI was used to counterstain the nuclei (Blue). Abbreviations: WT, wildtype; HET, heterozygous; KO, knockout; Am, ameloblasts; Od, odontoblasts; P, dental pulp; si, stratum intermedium; man, mandible; max, maxilla; ost, osteoblasts; trab, bone trabeculae; a, ameloblast with high number of PEX11b-stained peroxisomes; b, ameloblast with low number of PEX11b-stained peroxisomes.

We next investigated the distribution of PEX11b stained peroxisomes in the dental tissues. The presence or absence of the *Pex11b* gene in all animals used in this investigation was confirmed by genotyping DNA derived either from fresh tissue samples (3 WT and 3 HET mice bred in the animal facility in Giessen) or from FFPE-embedded tissue (1 WT, 1 HET and 4 KO mice obtained from the Johns Hopkins University [20,21]) (S1 Fig). PEX11b stained, roundly shaped and closely clustered peroxisomes were detectable in comparable amounts in ameloblasts, odontoblasts and in the cells of the stratum intermedium and of the dental pulp of the WT mice (Fig 2A and 2B). Ameloblasts contained variable amounts of PEX11b stained peroxisomes, hence, some contained high number while in others they were not detectable (Fig 2B, encircled cells, “a” and “b” respectively). This heterogeneity was not observed for the staining with the other peroxisomal markers used in this study. Previous studies have shown that *Pex11b* mRNA is highly expressed in the mandible of newborn mice [29]. However, the maxilla was not investigated [29]. Here we can confirm that PEX11b stained peroxisomes were particularly abundant (round structures) in the osteoblasts of the mandible as well as in the maxilla (alveolar bone) (Fig 2H and 2I). When observing the PEX11b staining in the dental cells of the *Pex11b* heterozygous mice, we found that the typical punctuated peroxisomal pattern visible in the wildtype was almost absent and that cytoplasmic background staining was increased (Fig 2C and 2D). Overall, the staining resembled more the one of the knockout than the one of the wildtype. Concordant with this, the mRNA abundance of *Pex11b* was strongly reduced in the heterozygote (Fig 2G). In the knockout there was an ulterior increment of cytoplasmic staining and complete loss of the peroxisomal PEX11b staining. As expected the samples from the *Pex11b* knockout first molars did not contain mRNA for *Pex11b* (Fig 2G). Similarly to what was observed in the dental cells, in the osteoblasts of the alveolar bone of the maxilla the abundance of peroxisomes (round structures) stained with PEX11b was lowered in the *Pex11b* heterozygous mice while the cytoplasmic background was increased (Fig 2J). In the knockout only cytoplasmic background staining was visible (Fig 2K). These results demonstrate the presence of PEX11b containing peroxisomes in all analyzed dental cell layers and confirm their reduction in the *Pex11b* heterozygous mice and their absence in the *Pex11b* knockout mice.

**Fig 3:**
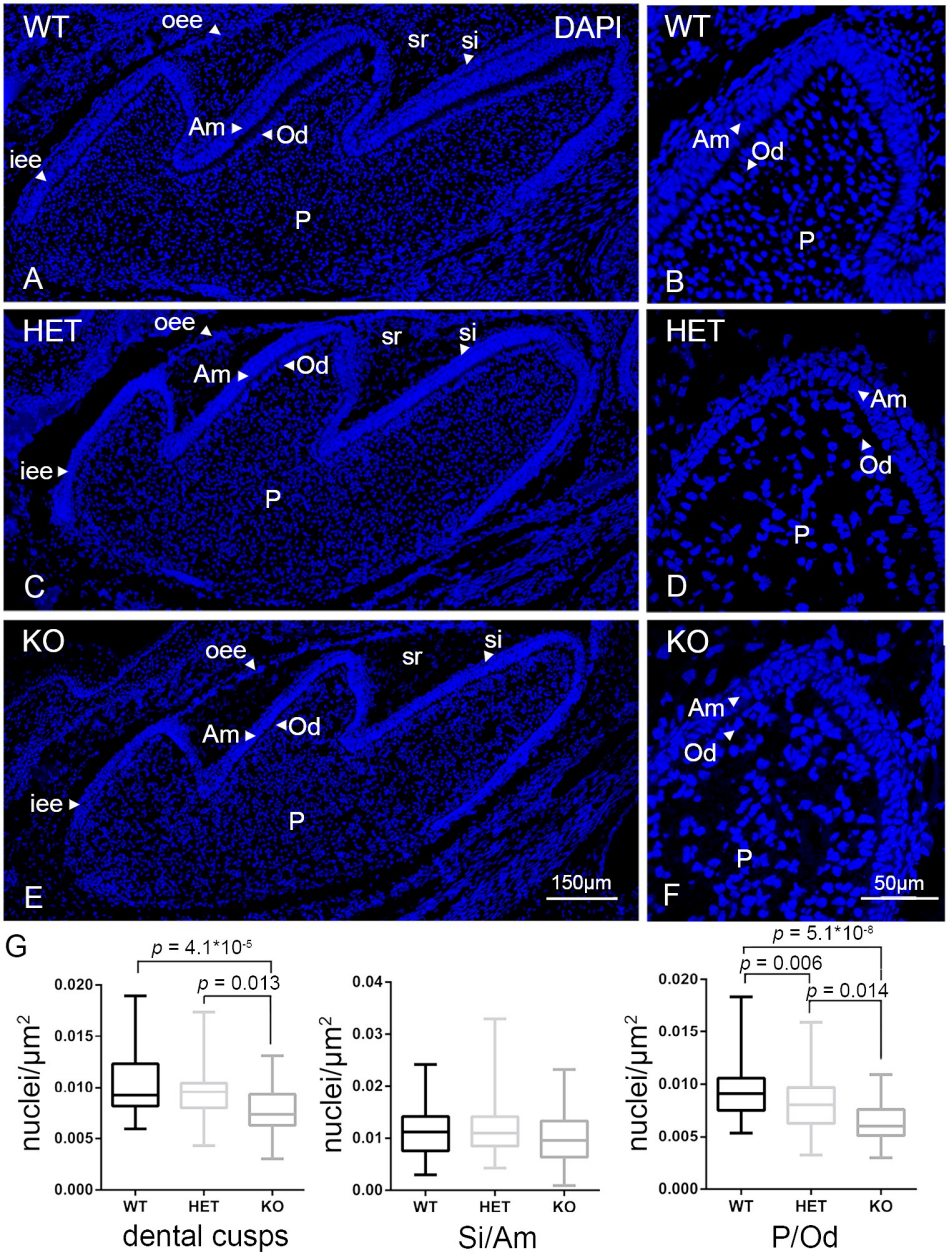
Fluorescent staining and morphometric analysis of the nuclei in developing first molars. (A-F): DAPI-stainings of the first molars of wildtype (WT) (A and B), heterozygous (HET) (C and D) and knockout mice (KO) (E and F): The pictures show the typical cell types during the bell stage of odontogenesis: Outer enamel epithelium (oee, arrowheads), stellate reticulum (sr), stratum intermedium (si, arrowheads), inner enamel epithelium / ameloblasts (iee / Am, arrowheads), odontoblasts (Od, arrowheads) and dental pulp (P). Bars shown in figures E and F represent the magnification for the corresponding image columns of all three genotypes. G: Statistical analysis of the number of nuclei/μm^2^ of the dental cusps, stratum intermedium/ameloblasts (Si/Am) and pulp/odontoblasts (P/Od) of first molars of wildtype (WT, black boxes), heterozygous (HET, light gray boxes) and knockout (KO, dark gray boxes) mice. The boxplots display minimum/maximum values, median and standard deviation obtained from the analyses of CLSM-images. Number (n) of evaluated CLSM images: n = 143. For statistical analyses and graph design GraphPad Prism 9 with Tukey-posthoc-test (one-way Anova) were used. α = 0.05.</FIGURE_CAPTION>

Nuclear DAPI-staining was next used to investigate the number of nuclei present in each layer (Fig 3A–3F). S4 Fig shows amplified cytoplasmic background signal to highlight the areas of the individual cells (gray) overlayed with the corresponding DAPI staining to improve differentiation of stratum intermedium and of ameloblast and odontoblast layers (S4A–S4C Fig).

Because of the difficulty of separating the individual dental cell layers in the apparently developmentally delayed knockout teeth we decided to evaluate the number of nuclei in the layer containing the ameloblasts/stratum intermedium cells (Si/Am), in the layer containing the odontoblasts/pulpa cells (P/Od) and in the whole dental cusp containing all layers (S2A Fig). As variations in dental sections orientations are inevitable when analyzing whole teeth due to their odd-shaped structure nuclei were counted only in longitudinally cut section areas and their number was normalized with the size of the ROI. This is especially important when analyzing teeth from different genotypes that appear to be developing differently with regards to their size and cell layer orientation. To avoid this bias for all the morphometric measurements in this paper we quantified only those areas that were mostly longitudinally cut as determined by the nuclear orientation. After evaluating several different images per tooth, genotype and mice, the morphometric analyses suggested a significant reduction of the number of the nuclei in the whole dental cusps (Fig 3G). The determining factor for this result was the layers of P/Od (Fig 3G).

### Differences in the immunofluorescence stainings for proliferation or apoptosis are difficult to interpret and need further experimental proves

**Fig 4:**
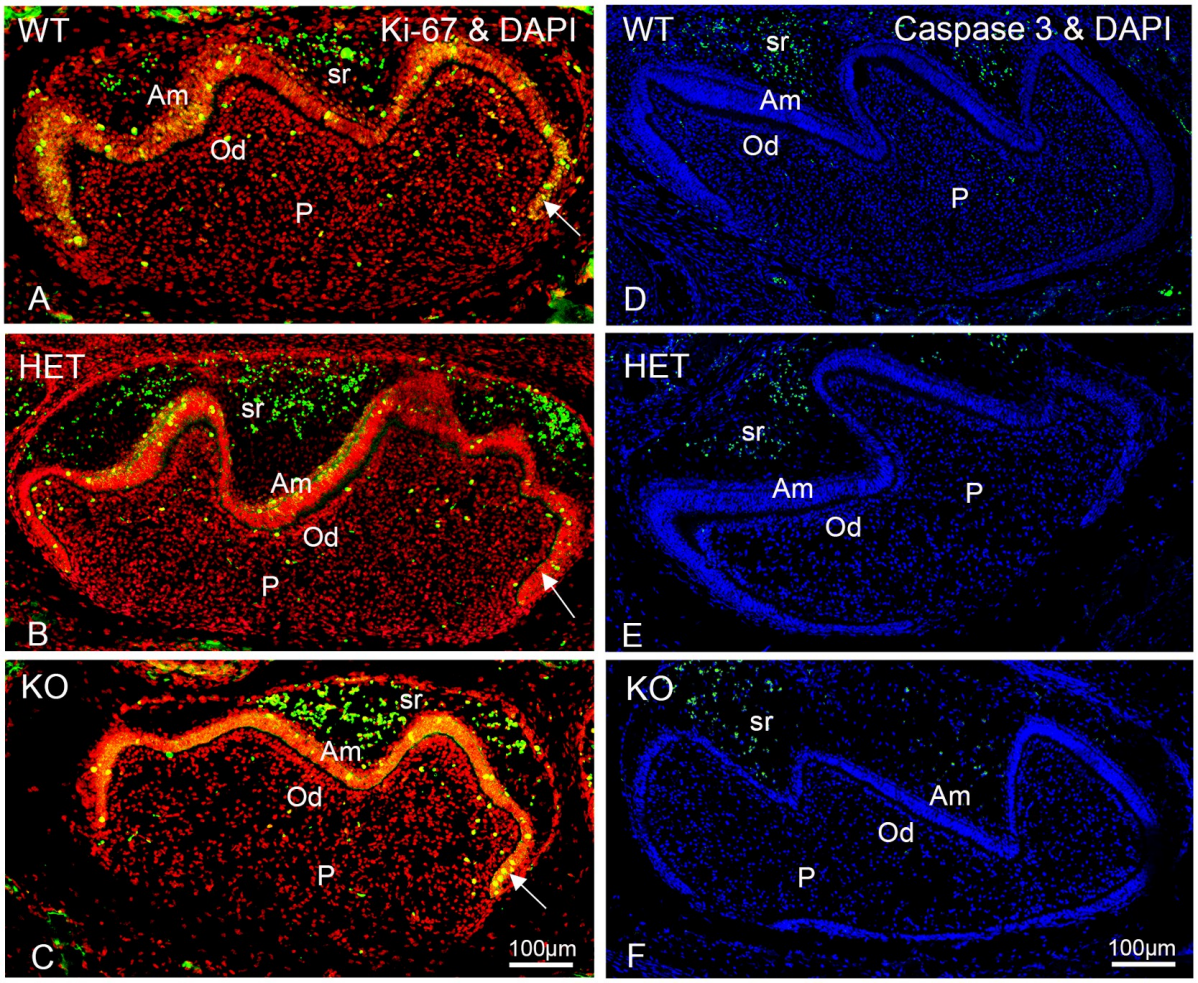
Differences in the immunofluorescence staining for Ki-67 and for activated caspase-3 are difficult to interpret and need further experimental proves. (A-F): Immunofluorescence analyses of wildtype (WT), heterozygous (HET) and knockout (KO) mouse molars in the bell stage using antibodies directed against Ki-67 (green fluorescence) (A-C) and activated caspase-3 (green fluorescence) (D-F) to highlight the number of mitotic and apoptotic cells, respectively. Bars shown in figures (C) and (F) represent the magnification for the corresponding image columns of all three genotypes. DAPI was used to counterstain nuclei. In (A-C) the DAPI staining is shown in red (false color instead of blue) for better visualization of the colocalization (yellow) with the green Ki-67 staining. Arrow: Inner enamel epithelium. Abbreviations: WT, wildtype; HET, heterozygous; KO, knockout; Am, ameloblasts; Od, odontoblasts; P, dental pulp; sr, stellate reticulum.

The morphometrically determined reduced number of nuclei suggested that the dental cell number was decreased when peroxisomes are dysfunctional. We hypothesized that this observation might be caused by less frequent mitotic events. To assess this, we used an antibody against Ki-67, a marker that is exclusively activated during mitosis. Ki-67-staining was particularly abundant at the inner enamel epithelium of the cervical loop (Fig 4A–4C, arrows) where actively dividing cells are located. We found no differences in the nuclear Ki-67-staining within odontoblasts and pulp cells of heterozygous and knockout mice. The morphometric analysis of the Ki-67-staining in the ameloblasts was rendered difficult by the strong cytoplasmic labelling, which was particularly intense in the knockout but could also be seen in the heterozygous teeth (Fig 4A–4C). No apparent difference was qualitatively observed in the abundance of Ki-67-staining in ameloblasts amongst the different genotypes. Moreover, particularly strong cytoplasmic Ki-67-labeling was detected in the cells of the stellate reticulum (Fig 4A–4C). Due to the unexpected Ki-67-staining pattern the immunofluorescence analysis remained inconclusive as to whether the lowered number of nuclei could be directly attributed to changes in the mitotic rate or not.

We also analyzed if increased apoptosis led to elevated cell loss in heterozygous and knockout teeth. To this purpose we stained the dental tissue using an antibody against the activated form of caspase 3, a typical marker for apoptotic cells. At this developmental stage no striking difference in the number of caspase 3-positive cells could be detected in either heterozygous or knockout teeth (Fig 4D–4F). The images rather suggest a decrease in apoptotic events in the stellate reticulum and the dental pulp of the knockout teeth (Fig 4F).

### The number of peroxisomes is reduced in the *Pex11b-*knockout mice in the different cell types of the first molars

**Fig 5:**
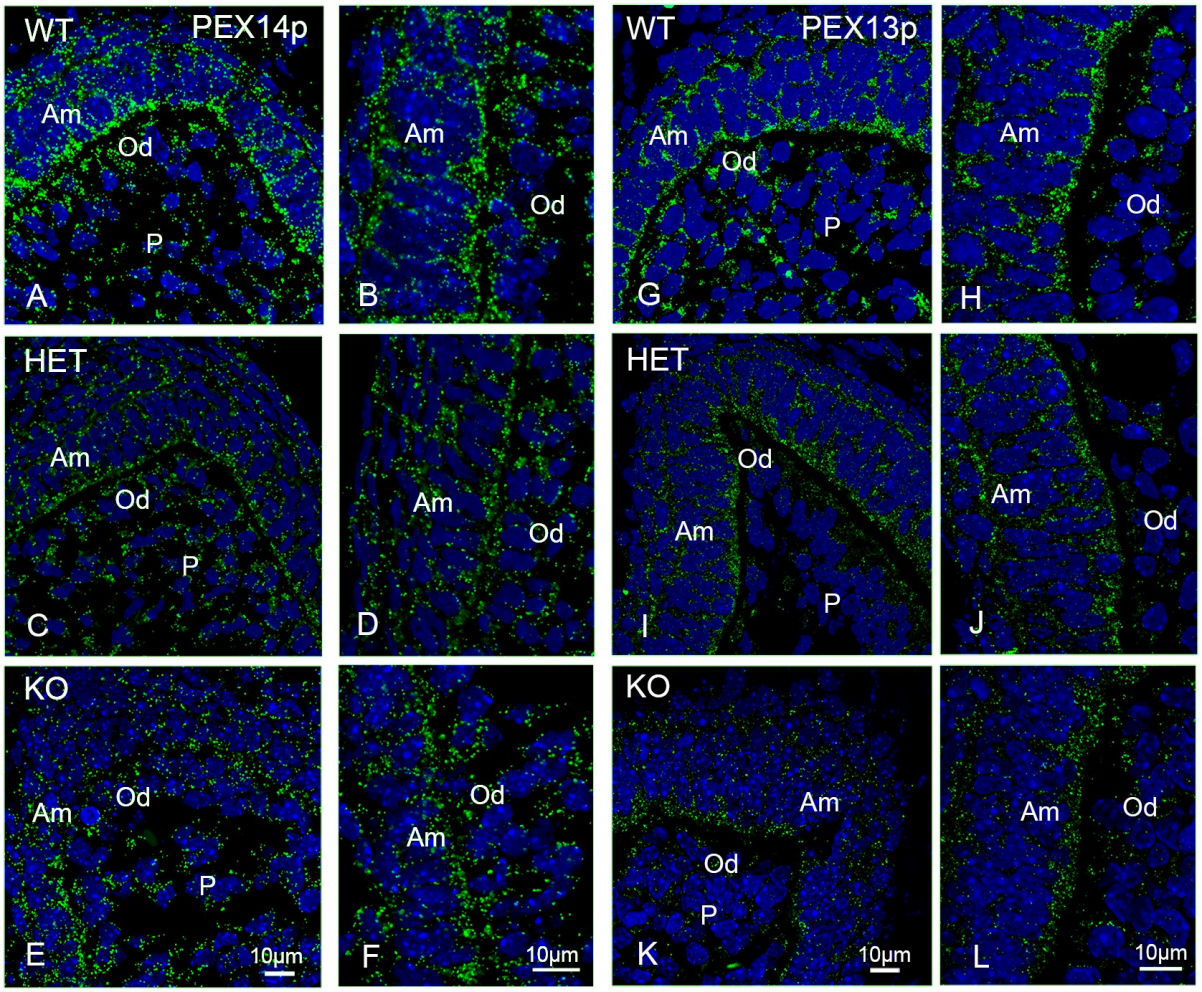
Immunofluorescence analyses of peroxisomes labelled with PEX14p and PEX13p in molars of wildtype, *Pex11b*-heterozygous and -knockout mice. (A-L): Molars of wildtype, *Pex11b*-heterozygous and -knockout mice were stained with antibodies directed against PEX14p (A-F) and PEX13p (G-L). Bars shown in figures (E) or (K) (overview images) and (F) or (L) (detailed images) represent the magnification for the corresponding image columns of all three genotypes. In all sections (A-L) DAPI was used to counterstain the nuclei (Blue). Abbreviations: WT, wildtype; HET, heterozygous; KO, knockout; Am, ameloblasts; Od, odontoblasts; P, dental pulp.

We wanted to investigate whether the knockout of PEX11b changes the number of peroxisomes in the dental cells of the first molars. To avoid possible biases during peroxisome counting due to differences in marker protein abundance we decided to use different antibodies to highlight the peroxisomal compartment during immunofluorescence analysis. Double staining with the PEX14p antibody and the marker proteins amelogenin (S5A–S5E Fig) and pancytokeratin (S5F–S5H Fig) for ameloblasts and vimentin (S5I–S5K Fig) for odontoblasts was used to colocalize the peroxisomal staining with the layers of the dental cells. PEX14p is currently one of the most suitable markers for the detection of peroxisomes as its abundance does not vary across different cell types allowing the comparative analysis of peroxisome abundance across different tissues independently of protein abundance [36]. As previously reported by our group [34], PEX14-stained peroxisomes are clearly detectable in all layers of the analyzed dental cells in the wild type but also in the heterozygous and knockout mice (S5A–S5K Fig). PEX14p and PEX13p are closely associated within the peroxisomal membrane and mediate the translocation of peroxisomal matrix proteins [37]. In wildtype animals, PEX14p- and PEX13p-labelled peroxisomes were more abundant in the ameloblasts than in the odontoblasts (Fig 5A, 5B, 5G and 5H). In ameloblasts and odontoblasts of heterozygous and knockout animals, the number of PEX14p- and PEX13p-stained peroxisomes was reduced compared to wildtype animals (Fig 5C–5F and 5I–5L).

**Fig 6:**
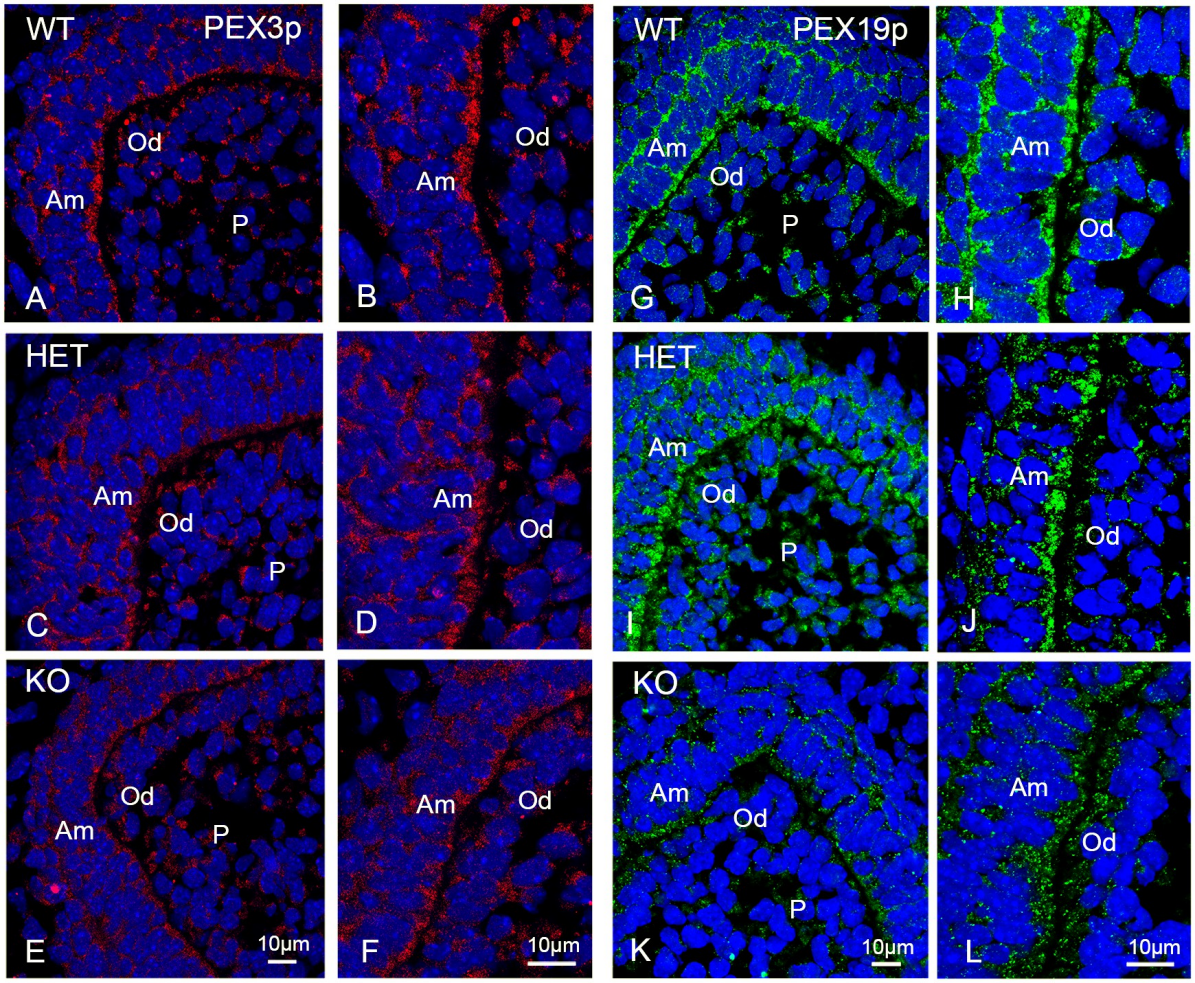
Immunofluorescence analyses of peroxisomes labelled with PEX3p and PEX19p in the molars of wildtype, *Pex11b*-heterozygous and -knockout mice. (A-L): Molars of wildtype, *Pex11b*-heterozygous and -knockout mice were stained with antibodies directed against PEX3p (A-F) and PEX19p (G-L). In all sections (A-L) DAPI was used to counterstain the nuclei (Blue). Bars shown in figures (E) or (K) (overview images) and (F) or (L) (detailed images) represent the magnification for the corresponding image columns of all three genotypes. Abbreviations: WT, wildtype; HET, heterozygous; KO, knockout; Am, ameloblasts; Od, odontoblasts; P, dental pulp.

Next, we analyzed peroxisomal number using antibodies directed against “early” peroxins involved in peroxisomal membrane biogenesis. Together with PEX3p, an integral peroxisomal membrane protein, the cytoplasmic shuttle receptor PEX19p, participates in peroxisomal membrane biogenesis [38]. PEX19p can be present either in the cytoplasm or on the peroxisomal membrane as well as in both locations, depending on the individual cell type [39]. Interestingly, in comparison to most other adult tissues, in teeth of new-born mice this protein was mainly located on the peroxisomal membrane and less in the cytoplasm (Fig 6G and 6H).

While the antibody directed against PEX19p (Fig 6G and 6H) provided a strong evaluable peroxisomal labelling, the staining of PEX3p was less intense (Fig 6A and 6B). In the molars of all three genotypes PEX19p-stained peroxisomes were more abundant in the ameloblasts than in the odontoblasts, and almost absent in the dental pulp cells (Fig 6G–6L). In the wildtype molars, PEX3p-stained peroxisomes could be visualized in both ameloblasts and odontoblasts (Fig 6A and 6B). The intensity of PEX19p- and PEX3p-stained peroxisomes was reduced in knockout molars (Fig 6A–6L). Since the individual peroxisomes stained with PEX3p were difficult to discern and—in the knockout teeth—were hardly distinguishable from the cytoplasmic background, morphometric analysis was not possible.

**Fig 7:**
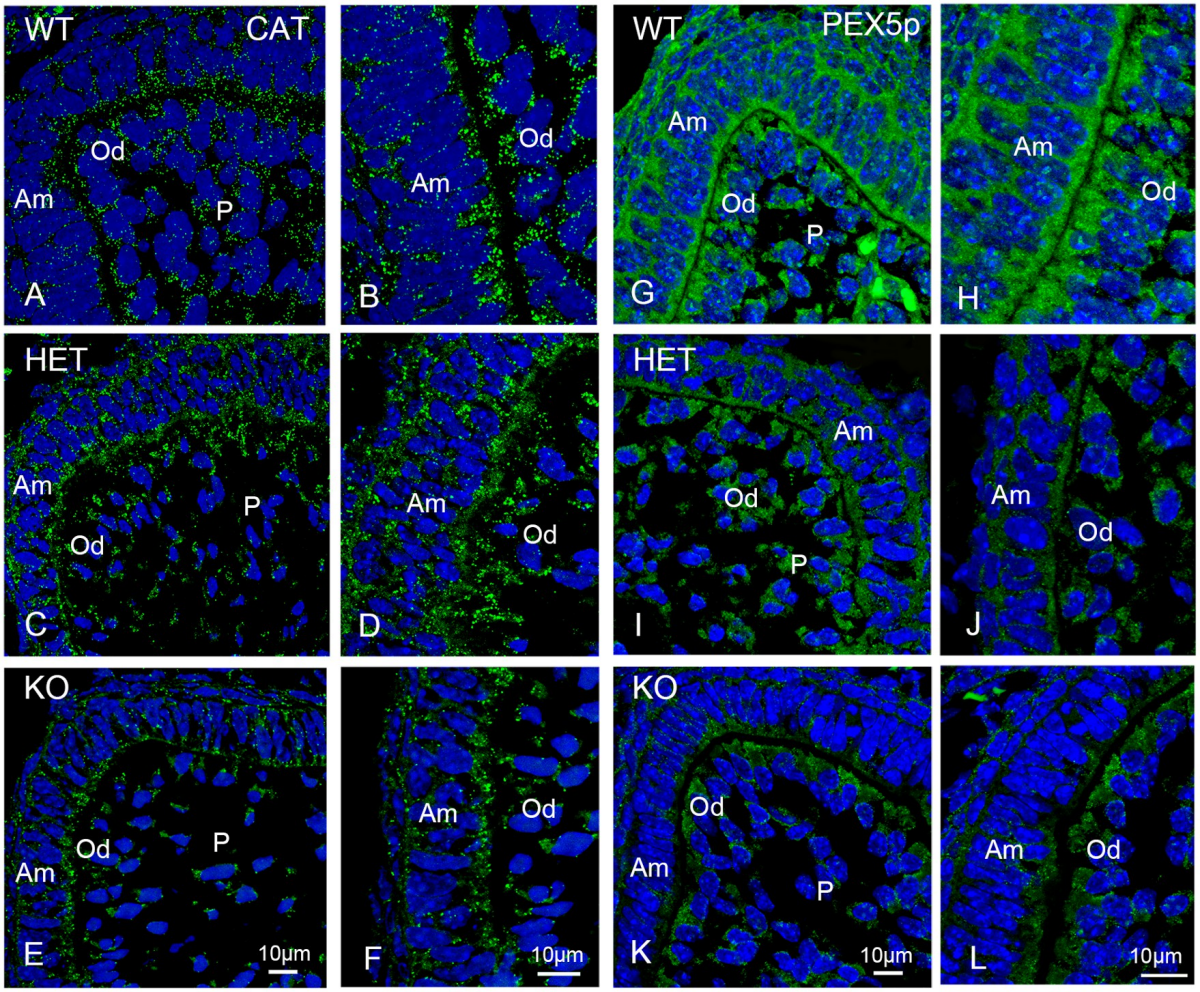
Immunofluorescence analyses of peroxisomes labelled with catalase (CAT), and PEX5p in the molars of wildtype, *Pex11b*-heterozygous and -knockout mice. (A-L): Molars of wildtype, *Pex11b*-heterozygous and -knockout mice were stained with antibodies directed against CAT (A-F) and PEX5p (G-L). Bars shown in figures (E) or (K) (overview images) and (F) or (L) (detailed images) represent the magnification for the corresponding image columns of all three genotypes. In all sections (A-L) DAPI was used to counterstain the nuclei (Blue). Abbreviations: WT, wildtype; HET, heterozygous; KO, knockout; Am, ameloblasts; Od, odontoblasts; P, dental pulp.

Next we investigated peroxisome abundance using antibodies directed against the matrix protein catalase and the peroxin PEX5p. Catalase is a peroxisomal enzyme that scavenges H_2_O_2_ and is the “historical” marker for peroxisomes [2,3]. PEX5 is a cytoplasmic shuttle receptor that is responsible for the recruitment and membrane translocation of peroxisomal matrix proteins [37]. An overview of the distribution of the staining of catalase and PEX5p in the first molars is shown in S6A–S6I and S7A–S7I Figs respectively. At higher magnification, it can be observed that in the ameloblasts and odontoblasts of wildtype mouse molars the catalase antibody produced the typical dot-like pattern for peroxisomes (Fig 7A and 7B).

In contrast, in the ameloblasts and odontoblasts of the heterozygous and the knockout animals, an increasingly diffuse, slightly cytoplasmic staining, with less distinct peroxisomes than in wildtype animals was observed for catalase (Fig 7C–7F). Overall, in these animals the number of catalase-stained peroxisomes was reduced. Moreover, the staining of individual peroxisomes was heterogeneous with some of the organelles appearing particularly bright and large (Fig 7C–7F). As expected, for PEX5p a strong cytoplasmic labelling was present in all cells of the molars (Fig 7G–7L). Like observed for the other investigated peroxins, the staining-intensity for PEX5p was diminished in the heterozygous and knockout molars. It is feasible that the loss of the cytoplasmic receptor PEX5p causes the observed cytoplasmic retention of catalase.

**Fig 8:**
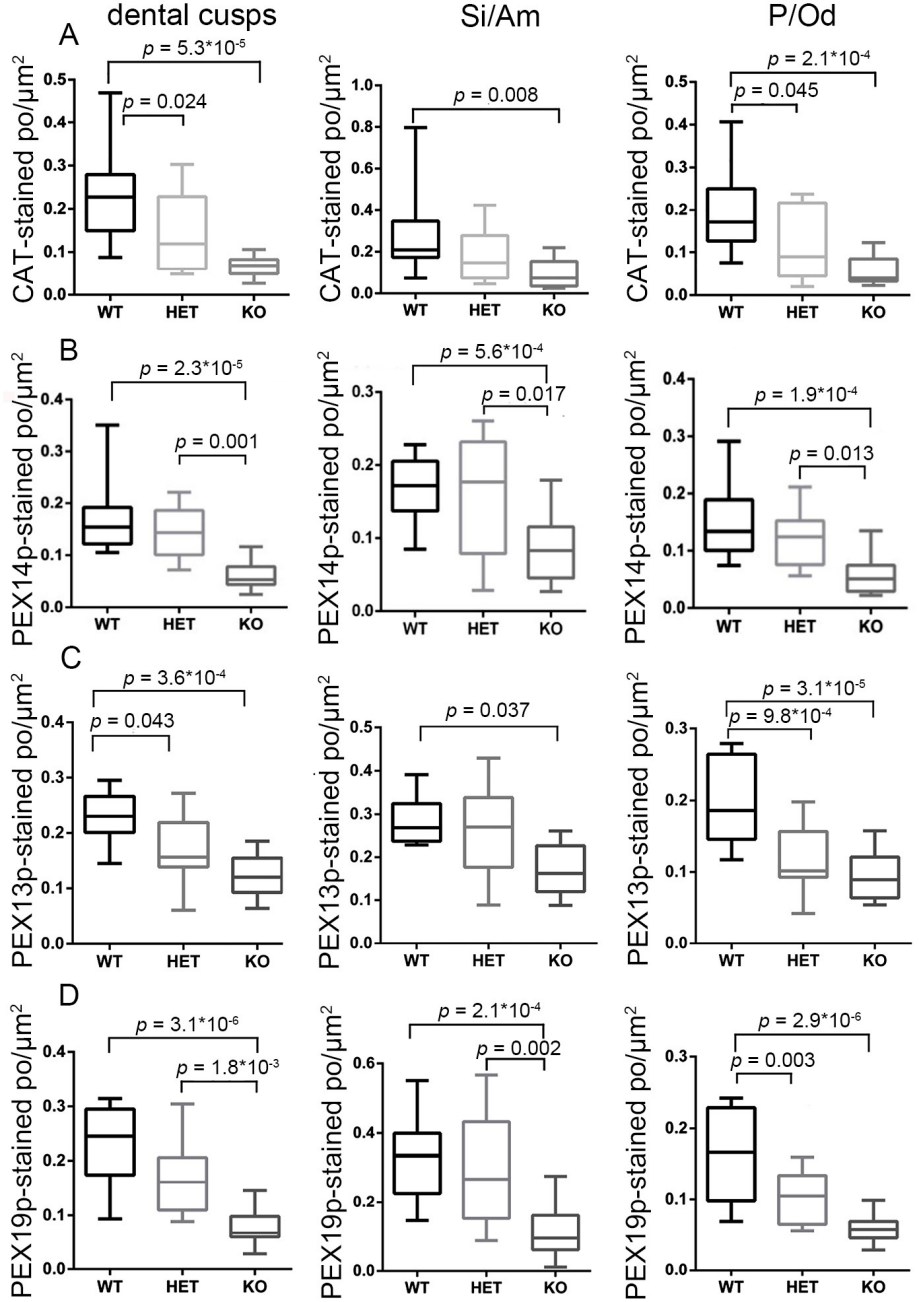
Abundance of peroxisomes in different regions of the developing first molars of wildtype, *Pex11b*-heterozygous and -knockout mice. (A-D): Graphical representation of the morphometric analysis of the peroxisomal number/μm^2^ in dental cusps, stratum intermedium/ameloblasts (Si/Am) and pulp/odontoblasts (P/Od) of first molars of wildtype (WT), heterozygous (HET) and knockout (KO) mice. The boxplots display minimum/maximum values, median and standard deviation obtained from the analysis of CLSM-images. Number (n) of evaluated CLSM images: n = 36. Y-axes were adjusted according to the scattering of the values and might therefore differ. For statistical analysis and graph design GraphPad Prism with Tukey-posthoc-test (one-way Anova) were used. α = 0.05.</FIGURE_CAPTION>

To accurately investigate peroxisomal numbers we performed morphometric analyses using the CLSM-images of PEX14p, PEX13p, PEX19p and catalase (Fig 8). The morphometric analyses confirmed that, in comparison to wild type molars, the number of peroxisomes in the knockout mice was significantly reduced to approximately half of the wildtype values in all areas of the first molar independently of the used antibodies (Fig 8). In the dental cusps of heterozygous molars only the number of catalase- and PEX13p stained peroxisomes was significantly decreased. A reduction of catalase-, PEX13p- and PEX19p-stained peroxisomes was observed in the P/Od-region of the heterozygous mice, while no significant change was measured for PEX14p-stained peroxisomes in these animals (Fig 8). In the Si/Am-region of the heterozygous mice no significant change of the peroxisomal number was observed (Fig 8).

### The knockout of *Pex11b* induces secondary mitochondrial disturbance and affects mitochondrial protein composition in odontoblasts and ameloblasts

It is commonly observed that when peroxisomes become dysfunctional mitochondrial morphology is also affected. To investigate the expanse of the mitochondrial compartment following the *Pex11b*-knockout we used two different mitochondrial marker proteins: Complex IV (subunit 1) a membrane associated component of the mitochondrial electron transport chain and SOD2, a mitochondrial matrix protein that is involved in ROS scavenging [40]. Both proteins have been previously shown to be affected when peroxisome are dysfunctional and are markers for mitochondrial alterations [22].

**Fig 9:**
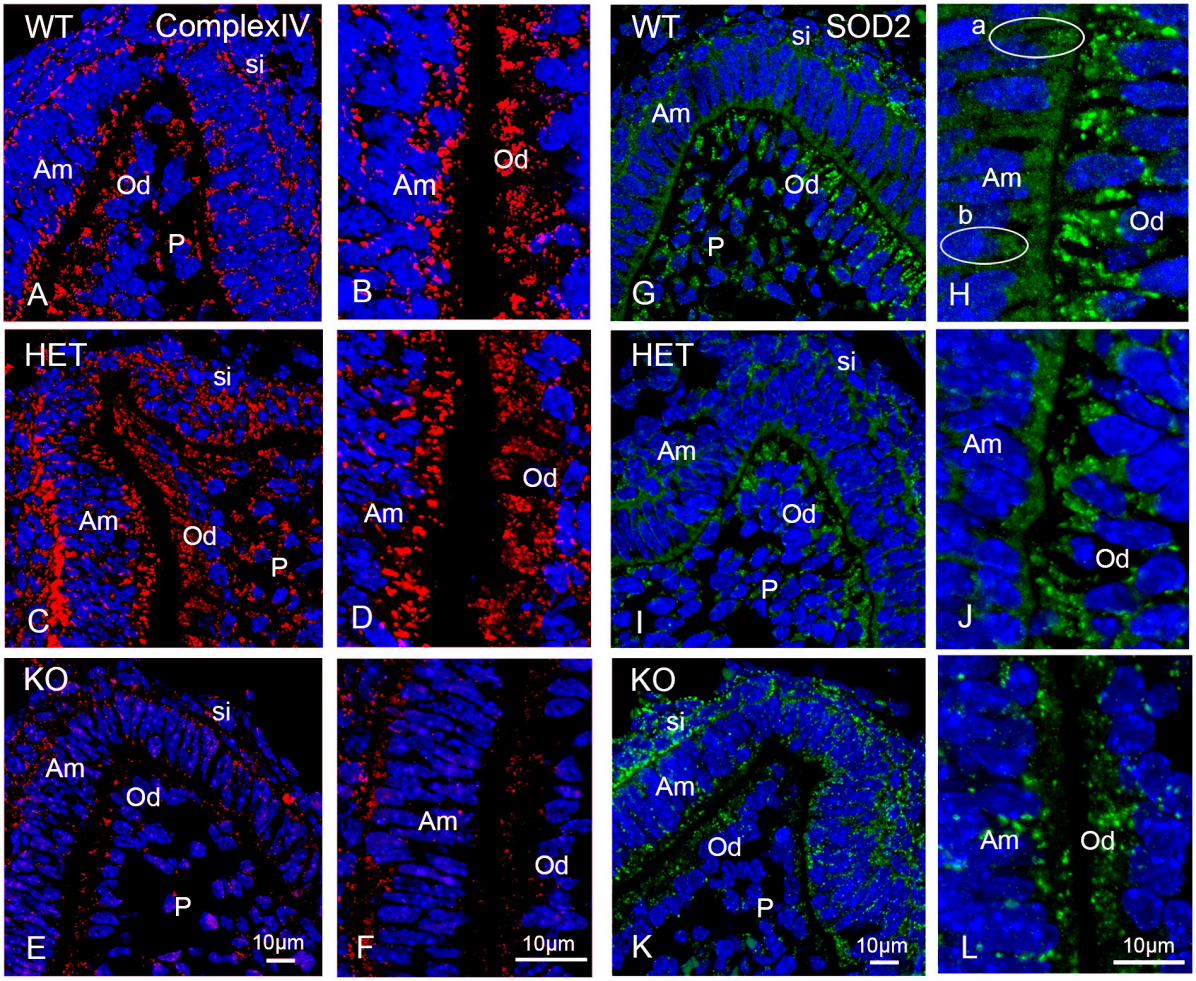
The peroxisomal defect caused by the PEX11b-deficiency induced secondary mitochondrial disturbance. (A-L): Immunofluorescence analyses of wildtype (WT), heterozygous (HET) and knockout (KO) mice bell stage molars using antibodies directed against complex IV of the mitochondrial respiratory chain (Complex IV) (A-F) and superoxide dismutase 2 (SOD2) (G-L). Bars shown in figures (E) or (K) (overview images) and (F) or (L) (detailed images) represent the magnification for the corresponding image columns of all three genotypes. DAPI was used to counterstain nuclei. Abbreviations: si, stratum intermedium; Am, ameloblasts; Od, odontoblasts; P, dental pulp. a, ameloblast with high number of SOD2-stained mitochondria; b, ameloblast with low number of SOD2-stained mitochondria.

Staining of the first molars of wildtype mice using an antibody against complex IV showed the typical mitochondrial pattern, visible as round or tubular structures of various sizes partially arranged in clusters. This mitochondrial pattern was clearly detectable in the stratum intermedium, ameloblasts, odontoblasts and the cells of the tooth pulp in wildtype mice (Figs 9A, 9B and S8A–S8D). In the heterozygous molars the staining of complex IV was reduced but still detectable in the ameloblasts and odontoblasts (Figs 9C, 9D and S8E–S8H). In contrast, in all dental cells of the knockout mouse molars the staining intensity for complex IV was nearly undetectable resulting in a much lower number of complex IV-positive mitochondria with respect to wildtype and heterozygous molars (Figs 9E, 9F and S8I–S8L).

We also used the antioxidative enzyme superoxide dismutase 2 (SOD2) as mitochondrial marker. SOD2 staining was detectable in the stratum intermedium, ameloblasts, odontoblasts and pulp cells of all genotypes (Figs 9G–9L and S9). In ameloblast the SOD2 staining was very heterogeneous, with some cells more intensively stained than others (longitudinally sectioned layers, Fig 9H, encircled cells, “a” and “b” respectively). In the heterozygous molars the overall abundance of SOD2-stained mitochondria in ameloblasts, odontoblasts and in the stratum intermedium cells was much lower than in wildtype molars (Figs 9I, 9J and S9A–S9F). In the knockout mice, the relative SOD2 staining was, unexpectedly, more intense in the stratum intermedium and odontoblasts than in heterozygous mice, and odontoblasts displayed less cytoplasmic staining (Figs 9I–9L and S9D–S9I). Individual ameloblasts showed high staining intensity, while the odontoblasts and cells of the pulp region displayed weaker SOD2-staining intensity than the wild type (Figs 9K, 9L and S9G–S9I). These results are a hint for changes in the mitochondrial compartment induced by the peroxisomal defect as was observed also in other tissues of general knockout mice with *Pex5* deficiency [22].

### Impact of the peroxisomal defect caused by the *Pex11b*-deficiency on the dental tissue differentiation of the first molars

**Fig 10:**
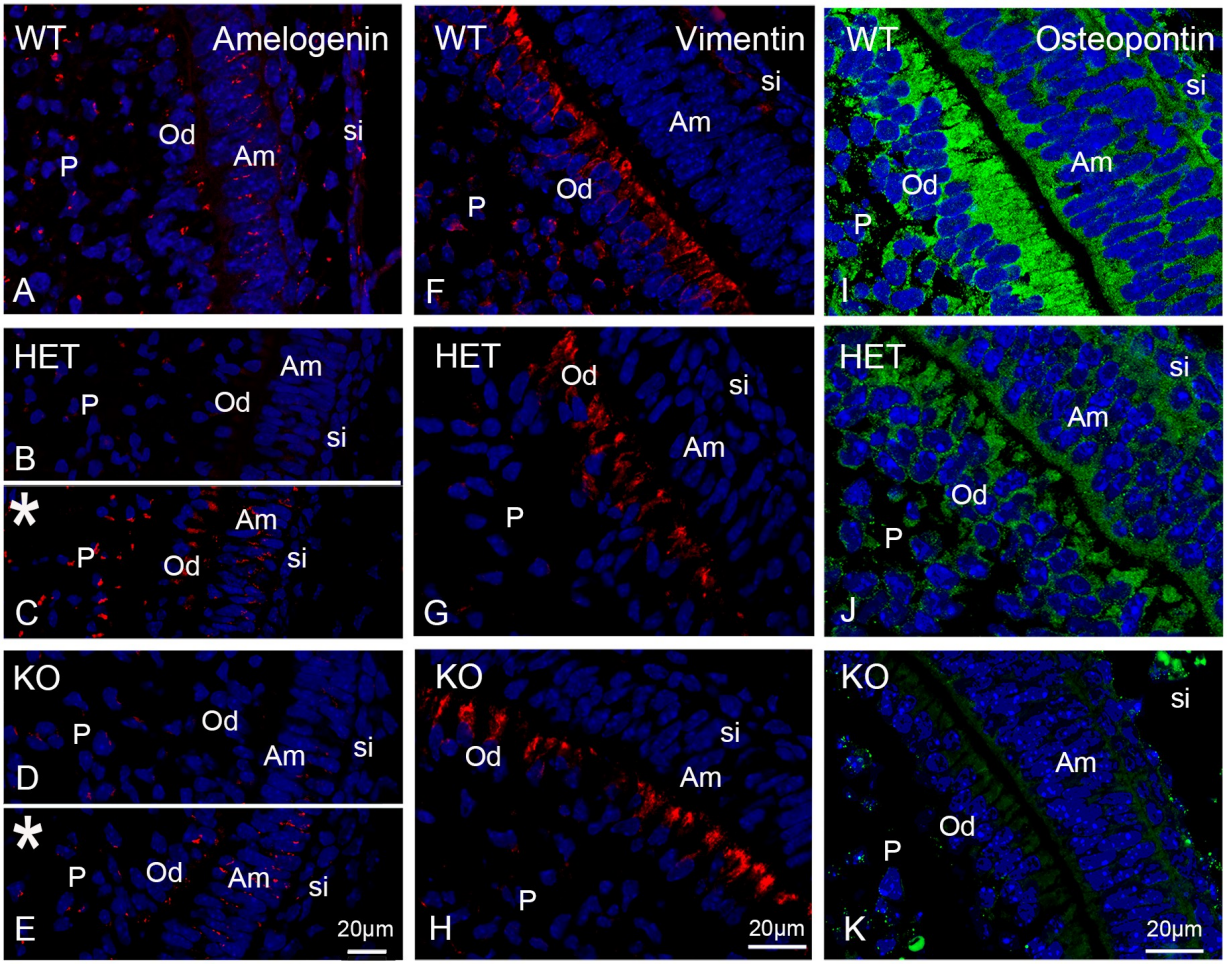
The intensity of the immunofluorescence staining for amelogenin, vimentin and osteopontin is lowered due to the peroxisomal dysfunction. Immunofluorescence analyses of amelogenin (A-E), vimentin (F-H) and osteopontin (I-K) in wildtype (WT), heterozygous (HET) and knockout (KO) mouse bell stage first molars. The pictures marked with an asterisk (*) represent the digitally intensified versions of (B) and (D). Bars shown in figures (E), (H) and (K) represent the magnification for the corresponding image columns of all three genotypes. DAPI was used to counterstain nuclei. Abbreviations: si, stratum intermedium, Am, ameloblasts; Od, odontoblasts; P, dental pulp.

The protein amelogenin is an excellent marker for ameloblasts and enamel. Amelogenin is the most important protein for enamel mineralization because it constitutes 80–90% of enamel proteins [41]. We therefore used an antibody against this protein to visualize the state of the enamel secretion in the first molars of mice. In addition to the enamel-related localization, amelogenin is also present in small amounts in odontoblasts and cells of the dental pulp [42,43]. Our immunofluorescence analysis of wildtype first molars showed that in ameloblasts the amelogenin staining was mostly concentrated and very strong at the secretory pole of the cells facing the enamel (Figs 10A and S10A–S10D). This staining pattern was previously observed for molars at P1 [44]. However, we also found amelogenin in the odontoblasts and pulp cells (Figs 10A and S10A–S10D). In the odontoblasts amelogenin staining was located in the developing Tomes fibers, the specific cellular processes of the odontoblasts positioned inside the dentin of adult teeth (Figs 10A and S10A–S10D). This localization was also previously described in an electron microscopy analysis of early-stage odontoblasts [45]. In the pulp cells, amelogenin staining appears located near the nucleus in a structure resembling the Golgi apparatus. In contrast to the wildtype, amelogenin staining intensity in heterozygous and knockout teeth was reduced (Figs 10B, 10D and S10E–S10L). Digital intensification of the red amelogenin signal by equal digital enhancement using Photoshop (Correction -> Levels -> Red Channel amplification) indicated that the labeling of ameloblasts in *Pex11b* heterozygous and knockout animals was proportionally much stronger reduced than the one for odontoblasts and pulp cells (Figs 10C, 10E, S10G, S10H, S10K and S10L). These results suggested disturbed tooth mineralization process in heterozygous and knockout animals.

We also tested the marker DSPP in our study, which, however, did not give satisfactory results for the identification of the odontoblast layer in the first molars on P0.5 mice. Therefore, to identify the odontoblast layer, we used the marker vimentin, a typical cytoskeletal element present in these cells [46]. In the wildtype, the apical and lateral sides as well as the processes of the odontoblast were strongly labeled using the antibody directed against vimentin (Figs 10F and S11A–S11C). This staining pattern was previously observed for incisor odontoblasts of rats and in molars of mice [34,47]. Additionally, also pulp cells were labelled although with less intensity (Fig 10F). In the odontoblasts of the heterozygote and the knockout the staining was exclusively concentrated to the apical part of the cells (Figs 10F–10H and S11D–S11I).

Osteopontin is an extracellular structural protein of bone matrix and was used as marker protein to further visualize differences in the mineralization process of the first molars due to the knockout of *Pex11b*. In the heterozygous teeth the osteopontin staining intensity was reduced compared to wildtype animals in all dental cell types and in the *Pex11b* knockout animals the staining was almost absent (Figs 10I–10K and S12A–S12L). We were also interested to see whether the observed developmental differences extended to the alveolar bone. Indeed, a decimation of osteopontin staining was evident in the heterozygous and more strongly in the knockout animals (S13A–S13C Fig).

We next wanted to investigate the distribution of osteocalcin in the first molars of the *Pex11b* knockout mouse. Osteocalcin is synthesized and secreted by odontoblasts into mineralizing dentin and by osteoblasts into the extracellular space of bone and is often used as mineralization marker for the bone matrix [48,49]. Unfortunately, the protein was below the detection limit for the immunofluorescence analysis in the first molars of all three genotypes, probably because these teeth are less advanced in their development in new-born mice (P0.5). It could, however, be detected in the incisors (more advanced in their development than the molars) and in the alveolar bone (S13D and S13I Fig). Strikingly, the osteocalcin staining was almost absent in *Pex11b*-heterozygous and -knockout incisors (S13D–S13H Fig). Like the staining for osteopontin, also the staining for osteocalcin was strongly reduced in the alveolar bone of heterozygous and knockout animals (S13I–S13K Fig).

**Fig 11:**
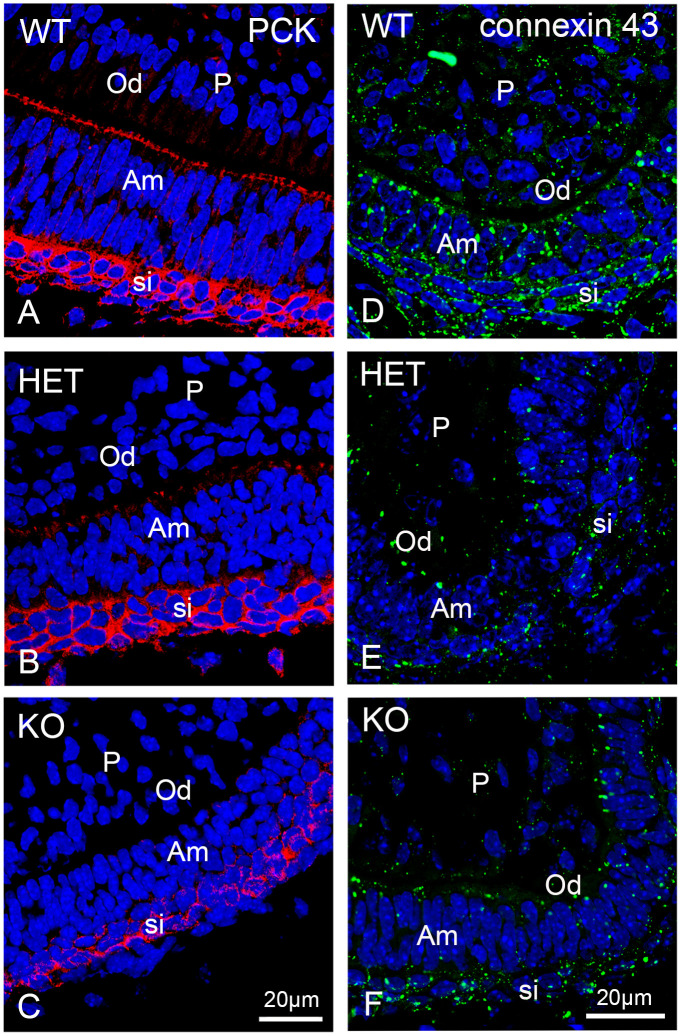
The intensity of connexin 43 and cytokeratin immunofluorescence staining is lowered when peroxisomes are dysfunctional. (A-C): Immunofluorescence analyses of cytokeratin (cytokeratin 5, 6 and 8. PCK) and (D-F): Connexin 43 wildtype (WT), heterozygous (HET) and knockout (KO) mouse bell stage first molars. Bars shown in figures (C) and (F) represent the magnification for the corresponding image row of all three genotypes. DAPI was used to counterstain nuclei. Abbreviations: si, stratum intermedium, Am, ameloblasts; Od, odontoblasts; P, dental pulp.

Proteins of the cytoskeleton play an important role for the differentiation and the secretory activity of dental cells [50,51]. Cytokeratins are intermediate filaments of epithelial cells and are thus present also in ameloblasts because these arise from the oral cavity epithelium. In S14 Fig an overview of the staining for pancytokeratin in the entire first molar can be observed. In wildtype mouse molars, the cells of the stratum intermedium showed the highest labelling intensity when using an anti-cytokeratin antibody detecting cytokeratins 5, 6 and 8 (Figs 11A and S14A–S14C). Odontoblasts and pulp cells displayed weaker staining (Fig 11A). Heterozygous mouse teeth exhibited much weaker labelling intensity at the secretory pole of the ameloblasts. In contrast, the staining of their stratum intermedium displayed a similar intensity as the one of wildtype teeth (Figs 11B and S14D–S14F). In the knockout mouse molars, the staining for cytokeratin at the secretory pole of the ameloblasts was almost indiscernible and the staining of the stratum intermedium was weaker in comparison to the other two genotypes (Figs 11C and S14G–S14I). Again, these obvious differences indicate retarded differentiation of the distinct dental cells.

Connexin 43 is a gap junction protein, which is present in ameloblasts, odontoblasts and cells of the stellate reticulum, stratum intermedium and dental papilla [52]. Connexin 43 is particularly relevant for ameloblast differentiation and enamel mineralization [53]. Albeit we observed connexin 43-labelling in all dental cell types of the first molars, the strongest labelling intensity was found in the cells of the stratum intermedium and in the ameloblasts of the wildtype animals, in which gap-junction plaques appeared larger and very abundant (Figs 11D and S15A–S15C). In odontoblasts and pulp cells the connexin 43 stained gap-junction plaques were clearly smaller and less abundant (Figs 11D and S15A–S15C). In heterozygous and knockout teeth, ameloblasts and cells of the stratum intermedium displayed a strongly reduced number of large connexin 43-labeled gap junction plaques (Figs 11E, 11F and S15D–S15I). In the odontoblasts and pulp cells of these teeth the gap junction plaques stained by connexin 43 were particularly small and much less abundant (Fig 11E and 11F).

## Discussion

Despite Steinberg and colleagues described enamel hypoplasia in Zellweger patients already three decades ago [9], until now only few investigations were made on how peroxisomal disorders affect tooth development. In 2004, a clinical study listed phenotypical features and pathologies of 31 long-lived (1.2–24 years old) Zellweger patients in detail [54]. In addition to organ defects these patients exhibited tooth associated pathologies such as dysgnathia, delayed eruption (*dentitio tarda*), malposition of the teeth and enamel hypoplasia [54]. Hypodontia and tooth malocclusions were also described in patients with the mildest form of PBDs, the infantile Refsum´s disease [15,19]. Another study from 2014 reported the oral manifestations and clinical management of a long-lived patient with mild Zellweger syndrome phenotype, but without focusing on the peroxisome-related molecular pathogenesis of the dental defects [17]. Nowadays more than 20 different diseases caused by mutations in more than 30 different genes including peroxins are included in the group of peroxisomal disorders [55]. These disorders can affect multiple organs systems but are usually associated with deficient peroxisomal ROS management and lipid metabolism [55].

### Delayed odontogenesis and peroxisomal loss in the PEX11b knockout mice

The knockout of *Pex11b* in mice causes a generalized peroxisomal defect in all cells of the body, which leads to not yet fully clarified, Zellweger-like, multiple organ dysfunctions, growth delay and tissue malformations [20,21]. Evidence for developmental retardation of the nervous system, the kidneys, the liver and of ossification were previously provided by other publications [20,21]. The extend of the developmental delay and organ malformations is so severe, that the pups die within the first day of life. We were therefore interested to investigate whether, in addition to the other already described organ defects associated with the severe peroxisomal dysfunction induced by the PEX11b knockout, also early odontogenesis was disrupted.

Here we provided for the first time evidential support that the absence of a peroxin caused the retardation of odontogenesis in mice. The severe peroxisomal defect caused by the PEX11b knockout was highlighted by the reduced number of peroxisomes present in ameloblasts, odontoblasts and cells of the stratum intermedium and pulpa. The dental cell layers also showed a reduced number of nuclei in the *Pex11b* knockout. As a possible consequence of these alterations, we suspected a delay in odontogenesis as observed by the disarrangement of the ameloblast and odontoblast layers in the *Pex11b* knockout compared to wild-type mice. In support of this hypothesis, we showed that several markers of tooth cell development, including the secretory proteins amelogenin, vimentin, osteopontin and osteocalcin, the intermediate filament cytokeratin and the gap junction protein connexin 43, were severely reduced in the dental layers following the *Pex11b* knockout.

The tissue disorganization resulting from the disruption of the metabolic function of peroxisomes in the absence of PEX11b is not restricted to dental cells. Indeed, developmental defects have been previously described in the brain, liver and kidneys of the same *Pex11b* knockout mice [21,28]. It is therefore likely that the observed dental phenotype is the result of general underdevelopment associated with severe peroxisomal defects of the Zellweger spectrum, for which *Pex11b* knockout mice are a valid model. Our observations add a novel component to the previously described spectrum of symptoms associated with severe peroxisomal defects.

### ROS as a possible cause of delayed odontogenesis

The observed loss of peroxisomes caused by the PEX11b knockout probably led to a secondary mitochondrial defect and increased ROS load in dental tissues. To counteract oxidative stress-induced damage mammalian peroxisomes harbor the enzymes catalase, superoxide dismutase (SOD1), peroxiredoxin 1 and glutathione S-transferase [56–58]. The loss of normal peroxisomal function therefore inevitably increases oxidative stress leading to dysregulation of normal cell function due to ROS-mediated DNA-damage, protein oxidation, cell-membrane disruption through lipid peroxidation, organelle autophagy or alterations to signaling pathways [59–62]. A commonly observed issue with peroxisomal dysfunction and increased ROS accumulation are aberrations of the mitochondrial structure (severe morphological alterations of cristae, membrane disruption and swelling, loss of normal mitochondrial function and increased mitochondrial autophagy) [22,63–65]. This problematic correlation exacerbates the cellular oxidative stress since mitochondrial dysfunction increments ROS production (“ROS induced ROS release”) [66,67].

The lowered respiratory chain complex IV-staining intensity that we have observed in the *Pex11b-*knockout mouse molars suggested a disturbance of mitochondrial function, typical for severe peroxisomal disorders. When PEX5p was deleted in mice the protein amount of the respiratory chain complexes I, III and IV were reduced in the liver [22]. In cells, hydrogen peroxide exposure and associated elevated ROS specifically led to the degradation of mitochondrial mRNAs including those encoding for cytochrome *c* oxidase (complex IV) subunits I and III as well as the ones for ATPase 6, NADH dehydrogenase 6, 16S rRNA and 12S rRNA [68].

Similar to cultured neocortex neurons derived from *Pex11b-*knockout mice [68], and to the hepatocytes of the *Pex5*-knockout mice [22] also in the ameloblasts and in the cells of the stratum intermedium of the *Pex11b-*knockout the abundance of SOD2-stained mitochondria was increased when compared to heterozygotes. Interestingly, cells of the stratum intermedium displayed particularly high SOD2 staining intensity in all genotypes. This cell layer plays a significant role in the maintenance of the transport of ions such as calcium from the serum to the adjacent ameloblasts [69,70]. These transport process require high amounts of ATP, which must be generated through mitochondrial oxidative phosphorylation. Increased activity of the respiratory chain is commonly associated with increased levels of oxidative stress which might be counteracted by the presence of antioxidative enzymes such as SOD2 [71].

A detrimental consequence of mitochondrial dysfunction is the loss of oxidative phosphorylation power with concomitant depletion of ATP synthesis. Indeed, in patients with X-ALD a severe defect of the respiratory chain in mitochondria of spinal cord cells was observed [72]. The mitochondrial dysfunction exacerbated the cellular redox imbalance and led to extremely elevated ROS-production with concomitant oxidation of TCA-cycle and glycolytic enzymes and decreased ATP levels [72]. Since odontogenesis is a high energy consuming process, secondary mitochondrial dysfunction caused by peroxisomal defects might contribute to the retardation of tooth development in PBD-patients. In dental tissue several lines of evidence show that elevated ROS, like the one occurring when peroxisomes are deficient, interferes with the function of ameloblasts and odontoblasts and disrupts the mineralization of teeth [73]. Indeed, during fluorosis, a condition that leads to mineralization defects, increased exposure to fluoride concentrations caused oxidative stress and mitochondrial damage in ameloblasts [74–76]. This led to the activation of UCP2, an electron transport chain uncoupler with concomitant decreased ATP production and loss of ameloblast function [74].

### Structural alterations are present in the dental cell layers of the PEX11b knockout mice

A protein affected by oxidative stress and altered in peroxisomal disorders is connexin 43 [77–79]. Connexin 43 is the main component of gap junctions and is required for tissue development, signaling and structural maintenance [80,81]. Connexin 43 silencing in dental pulp cells resulted in reduced expression of dentin sialophosphoprotein making it an important factor during odontoblast differentiation and arrangement [82]. The knockout of connexin 43 in mice, induced reduced mineral density in the enamel as well as ossification disorders with defects in the craniofacial region [53,83,84]. Also, humans suffering from a connexin 43 mutation display craniofacial deformities, hypomineralization of enamel and dentin [85], tooth malocclusion and microdontia [86]. Indeed, the differentiation level of the dental cells is directly proportional to the abundance of connexin 43 [52,87,88]. In our study, connexin 43 was abundantly located at the secretion pole and the lateral side of the ameloblasts but was distributed along the whole membrane in stratum intermedium cells. This agrees with previous immunohistochemical evaluations of rat teeth [87,88]. The staining intensity of apical and lateral connexin 43 was strongly reduced in *Pex11b* knockout mice hinting to disturbance of the intercellular connection and interaction.

The phosphorylation of connexin 43 is a prerequisite for the assembly of gap junctions [89]. Dephosphorylation of connexin 43 was observed in cardiac muscle and brain cells following ischemia-induced oxidative stress, ultimately leading to loss of gap junctions and cell-death [78,79,90]. Therefore, increased ROS-formation due to dysfunctional peroxisomes could lead to connexin 43 oxidation resulting in loss of gap junctions and disturbed intercellular communication of dental cells during odontogenesis. Moreover, dysfunctional plasmalogen synthesis in peroxisomes might affect connexin 43 protein levels due to disturbed composition of plasma membrane lipids as shown in retinal Müller cells [91]. In these cells, the siRNA-mediated knockdown of the mRNA encoding glycerone-phosphate O-acyltransferase (GNPAT), the content of plasmalogens was reduced to half causing a critical decrease of the connexin 43 expression. This resulted in reduced calcium-based gap junction intercellular communication as well as a migration defect [91]. It is feasible that also in dental cells reducing the abundance of connexin 43 might cause intercellular miscommunication and failure to correctly propagate and align during odontogenesis.

The severe peroxisomal defect caused by the knockout of *Pex11b* decreased staining intensity of amelogenin, osteocalcin, and osteopontin. Also, ossificated bone trabeculae surrounding the developing tooth appeared thinner in the knockout mice. We therefore suggest that functional peroxisomal metabolism supports odontogenesis and timely secretion of enamel-, dentin- and bone-matrix proteins during fetal development. The mineralization process of hard tissues exhibits some similarities resulting in cross-influence of the individual processes [92]. For example, amelogenesis imperfecta patients might also suffer skull deformities [93], similar to those observed in patients with Zellweger syndrome spectrum disorder [94]. In the enamel, growth and size of the hydroxyapatite crystals are mostly regulated by amelogenin [95] explaining why amelogenin-knockout mice possess an irregular enamel structure with increased roughness [30]. In previous studies the loss of amelogenin, similar to the decreased amelogenin staining intensity we observed here for the knockout mouse teeth, resulted in enamel hypomineralization and hypoplastic amelogenesis imperfecta [96–99]. Also, the secretion process of dentin (and bone matrix) appeared to be delayed when peroxisomes are dysfunctional as shown using the markers osteocalcin and osteopontin. Osteopontin belongs to the so-called SIBLINGS (Small Integrin-Binding LIgand, N-linked GlycoproteinS), which are crucial for crystal formation in osteoid and predentin [100]. In comparison to osteopontin, osteocalcin is mainly detectable in more differentiated odontoblasts and in predentin. This could account for the very weak staining in the developmentally delayed first molars of the *Pex11b* knockout mice. We found reduced staining intensity for osteopontin and osteocalcin also in the alveolar bone. Both osteoid and predentin are mainly composed of collagen type 1 and are secreted by osteoblasts and odontoblasts, respectively [101]. Because the mechanisms of mineralization of osteoid to bone and predentin to dentin are comparable, its defects result in hypomineralization of both hard tissues [102].

Since the general *Pex11b* knockout mice die within the first day after birth, comparative studies on the dentin and enamel amounts and general mineralization state of more developed teeth will only be possible in future studies with hard tissue specific *Pex11b* knockout mice.

## Supporting information

S1 File Tables of the used antibodies. Supplementary Table S1: List of primary antibodies used for the immunofluorescence analysis. Supplementary Table S2: List of secondary antibodies used for the immunofluorescence analysis.
(DOCX)

S1 Fig Genotyping of the PEX11b mice. (A) Schematic representation of the PEX11b WT locus and KO locus (both present in the HET) showing the hybridization site of the primers P8, P9 and PNeo used for genotyping PCR. Size of the expected PCR fragments are indicated. (B) Schematic representation of the results expected when conducting a multiplex PCR on the PEX11b mice samples. For WT only the 590 bp band should appear, for HET both the 590 and the 980 bp bands and for the KO only the 980 bp band. (C) Individual PCR reactions for the genotyping of the WT and HET mice bred and embedded at the JLU just prior to this study. For these DNA samples PCR using either the primer combination P8 and P9 or P8 and PNeo were used to determine the presence of the WT and the KO gene locus. All probes show the expected bands. (D) Multiplex PCR of the DNA samples obtained from the PEX11b sections embedded in 2002. All probes show the expected bands.
(TIF)

S2 Fig Morphometric analysis of nuclei and peroxisomes. Representation of the steps involved during morphometric analysis of the number of nuclei and peroxisomes. (A): The number of the nuclei (nuclei/μm^2^) was determined using the “Multi-point” tool of image J for manual counting after selecting and measuring the ROI. (B): The number of peroxisomes was analyzed using the automatic “particle counting” function of Image J. Steps 1.-3.: To distinguish the organelles from the background, the images were converted into 8-bit images and then the threshold value was set. Step 4.: Individual peroxisomes that appeared to be fused were separated by converting the image to binary and applying watershed. Steps 5. And 6.: After setting the scale (μm) and selecting the ROI all particles with intensities above the threshold value and with a size range of 0.01–1 μm^2^ were automatically counted by image J.
(TIF)

S3 Fig Microdissection of molars for RNA isolation. Exemplary image of microdissected tooth prior (Step 1.) and after (Step 2.) microdissection. The red outline filled with cyan background indicated the area of dissected tissue. The orange line indicates the stellate reticulum/ameloblast ameloblast border. The yellow line indicates the ameloblast/odontoblast border. Abbreviations: sr, stellate reticulum, Am, ameloblasts; Od, odontoblasts; P, dental pulp.
(TIF)

S4 Fig Fluorescent staining of the nuclei in developing first molars. (A-C): DAPI-stainings of the first molars of wildtype (WT) (A), heterozygous (HET) (B) and knockout mice (KO) (C): The pictures show the typical cell types during the bell stage of odontogenesis: Outer enamel epithelium (oee, arrowheads), stellate reticulum (sr), stratum intermedium (si, arrowheads), inner enamel epithelium / ameloblasts (iee / Am, arrowheads), odontoblasts (Od, arrowheads) and dental pulp (P). The cytoplasmic background present in the images of Fig 3 was digitally amplified to highlight the areas of the individual cells (gray) and overlayed with the corresponding DAPI staining.
(TIF)

S5 Fig Colocalization of the peroxisomal marker PEX14p with amelogenin, pancytokeratin and vimentin. Immunofluorescence analyses of the peroxisomal marker PEX14b and amelogenin (A-E), pancytokeratin (F-H) and vimentin (I-K) in wildtype (WT), heterozygous (HET) and knockout (KO) mouse bell stage first molars. The pictures marked with an asterisk (*) represent the digitally intensified versions of (B) and (D). Bars shown in figures (E), (H) and (K) represent the magnification for the corresponding image columns of all three genotypes. DAPI was used to counterstain nuclei. Abbreviations: si, stratum intermedium, Am, ameloblasts; Od, odontoblasts; P, dental pulp.
(TIF)

S6 Fig Overview of the catalase immunofluorescence staining. (A-I): Immunofluorescence analyses of catalase in wildtype (WT), heterozygous (HET) and knockout (KO) mouse bell stage first molars. Images B, E and H reflect catalase-stained images A, D and G respectively without the DAPI staining. Images C, F and I show higher magnification of the central dental cusp shown in A, D and G respectively. The bar shown in figure (H) indicates magnification for images A, B, D, E, G and H. The bar shown in Figure (I) represents the magnification the corresponding staining of all three genotypes. DAPI was used to counterstain nuclei. Abbreviations: si, stratum intermedium, Am, ameloblasts; Od, odontoblasts; P, dental pulp.
(TIF)

S7 Fig Overview of the PEX5 immunofluorescence staining. (A-I): Immunofluorescence analyses of PEX5 in wildtype (WT), heterozygous (HET) and knockout (KO) mouse bell stage first molars. Images B, E and H reflect PEX5-stained images A, D and G respectively without the DAPI staining. Images C, F and I show higher magnification of the central dental cusp shown in A, D and G respectively. The bar shown in figure (H) indicates magnification for images A, B, D, E, G and H. The bar shown in Figure (I) represents the magnification the corresponding staining of all three genotypes. DAPI was used to counterstain nuclei. Abbreviations: si, stratum intermedium, Am, ameloblasts; Od, odontoblasts; P, dental pulp.
(TIF)

S8 Fig Overview of the complex IV immunofluorescence staining. (A-L): Immunofluorescence analyses of complex IV in wildtype (WT), heterozygous (HET) and knockout (KO) mouse bell stage first molars. Images B, F and J reflect complex IV-stained images A, E and I respectively without the DAPI staining. Images C, G and K show higher magnification of the central dental cusp shown in A, E and I respectively. Images D, H and L show higher exposure of the central dental cusp shown in C, G and K respectively. The bar shown in figure (J) indicates magnification for images A, B, E, F, I and J. The bar shown in Figure (L) represents the magnification for the corresponding staining of C, D, G, H, K and L. DAPI was used to counterstain nuclei. Abbreviations: si, stratum intermedium, Am, ameloblasts; Od, odontoblasts; P, dental pulp; * higher exposure.
(TIF)

S9 Fig Overview of the SOD2 immunofluorescence staining. (A-I): Immunofluorescence analyses of SOD2 in wildtype (WT), heterozygous (HET) and knockout (KO) mouse bell stage first molars. Images B, E and H reflect SOD2-stained images A, D and G respectively without the DAPI staining. Images C, F and I show higher magnification of one of the dental cusps shown in A, D and G respectively. The bar shown in figure (H) indicates magnification for images A, B, D, E, G and H. The bar shown in Figure (I) represents the magnification for the corresponding staining of all three genotypes. DAPI was used to counterstain nuclei. Abbreviations: si, stratum intermedium, Am, ameloblasts; Od, odontoblasts; P, dental pulp. a, ameloblast with high number of SOD2-stained mitochondria; b, ameloblast with low number of SOD2-stained mitochondria.
(TIF)

S10 Fig Overview of the amelogenin immunofluorescence staining. (A-L): Immunofluorescence analyses of amelogenin in wildtype (WT), heterozygous (HET) and knockout (KO) mouse bell stage first molars. Images B, F and J reflect amelogenin-stained images A, E and I respectively without the DAPI staining. Images C, G and K show higher magnification of one of the dental cusps shown in A, E and I respectively. Images D, H and L show higher exposure of the dental cusp shown in C, G and K respectively. The bar shown in figure (J) indicates magnification for images A, B, E, F, I and J. The bar shown in Figure (L) represents the magnification for the corresponding staining of C, D, G, H, K and L. DAPI was used to counterstain nuclei. Abbreviations: si, stratum intermedium, Am, ameloblasts; Od, odontoblasts; P, dental pulp; * higher exposure.
(TIF)

S11 Fig Overview of the vimentin immunofluorescence staining. (A-I): Immunofluorescence analyses of vimentin in wildtype (WT), heterozygous (HET) and knockout (KO) mouse bell stage first molars. Images B, E and H reflect vimentin-stained images A, D and G respectively without the DAPI staining. Images C, F and I show higher magnification of the central dental cusp shown in A, D and G respectively. The bar shown in figure (H) indicates magnification for images A, B, D, E, G and H. The bar shown in Figure (I) represents the magnification for the corresponding staining of all three genotypes. DAPI was used to counterstain nuclei. Abbreviations: si, stratum intermedium, Am, ameloblasts; Od, odontoblasts; P, dental pulp.
(TIF)

S12 Fig Overview of the osteopontin immunofluorescence staining. (A-L): Immunofluorescence analyses of osteopontin in wildtype (WT), heterozygous (HET) and knockout (KO) mouse bell stage first molars. Images B, F and J reflect osteopontin-stained images A, E and I respectively without the DAPI staining. Images C, G and K show higher magnification of one of the dentals cusp shown in A, E and I respectively. Images D, H and L show higher exposure of the dental cusps shown in C, G and K respectively. The bar shown in figure (J) indicates magnification for images A, B, E, F, I and J. The bar shown in Figure (L) represents the magnification for the corresponding staining of C, D, G, H, K and L. DAPI was used to counterstain nuclei. Abbreviations: si, stratum intermedium, Am, ameloblasts; Od, odontoblasts; P, dental pulp; * higher exposure.
(TIF)

S13 Fig The staining intensity of osteocalcin and osteopontin is drastically reduced in incisors and alveolar bone after the knockout of *Pex11b*. (A-C): Immunofluorescence analyses of osteopontin in alveolar bone in wildtype (WT), heterozygous (HET) and knockout (KO) mice alveolar bone. (D-K): Immunofluorescence analysis of osteocalcin in wildtype (WT), heterozygous (HET) and knockout (KO) mice bell stage incisors (D-H) and alveolar bone (I-K). Bar shown in figure K represents the magnification for all images. DAPI was used to counterstain nuclei. The pictures marked with an asterisk (*) represent higher exposures of the images shown in “E” and “G”. Abbreviations: Am, ameloblasts; Od, odontoblasts.
(TIF)

S14 Fig Overview of the pancytokeratin immunofluorescence staining. (A-I): Immunofluorescence analyses of pancytokeratin in wildtype (WT), heterozygous (HET) and knockout (KO) mouse bell stage first molars. Images B, E and H reflect pancytokeratin-stained images A, D and G respectively without the DAPI staining. Images C, F and I show higher magnification of one of the dental cusps shown in A, D and G respectively. The bar shown in figure (H) indicates magnification for images A, B, D, E, G and H. The bar shown in Figure (I) represents the magnification for the corresponding staining of all three genotypes. DAPI was used to counterstain nuclei. Abbreviations: si, stratum intermedium, Am, ameloblasts; Od, odontoblasts; P, dental pulp.
(TIF)

S15 Fig Overview of the connexin 43 immunofluorescence staining. (A-I): Immunofluorescence analyses of connexin 43 in wildtype (WT), heterozygous (HET) and knockout (KO) mouse bell stage first molars. Images B, E and H reflect connexin 43-stained images A, D and G respectively without the DAPI staining. Images C, F and I show higher magnification of one of the dental cusps shown in A, D and G respectively. The bar shown in figure (H) indicates magnification for images A, B, D, E, G and H. The bar shown in Figure (I) represents the magnification for the corresponding staining of all three genotypes. DAPI was used to counterstain nuclei. Abbreviations: si, stratum intermedium, Am, ameloblasts; Od, odontoblasts; P, dental pulp.
(TIF)
