# Supplementary material for: Peroxisomal dysfunction interferes with odontogenesis and leads to developmentally delayed teeth and defects in distinct dental cells in Pex11b-deficient mice
Source: PLoS One. 2024 Dec 9;19(12):e0313445. doi: 10.1371/journal.pone.0313445 (PMC11627416; doi:10.1371/journal.pone.0313445)
Supplement: S1 File — Supplementary Table S1: List of primary antibodies used for the immunofluorescence analysis. Supplementary Table S2: List of secondary antibodies used for the immunofluorescence analysis. (DOCX) [file pone.0313445.s001.docx]

**MATERIALS AND METHODS**

**Supplementary Table S1:** primary antibodies

| antigen | Host species | dilution | source and year of acquisition | Ordering Number |
| --- | --- | --- | --- | --- |
| Catalase | rabbit | 1:4000 | Denis Crane, 2016 | - |
| PEX3p | rat | 1:100 | Colasante, 2017 | - |
| PEX5p | rabbit | 1:50 | Steve Gould, 2005 | - |
| PEX11Bp | rabbit | 1:200 | Antibodies online, 2024 | ABIN2626735 |
| PEX13p | rabbit | 1:500 | Denis Crane, 2014 |  |
| PEX14p | rabbit | 1:1000 | Denis Crane, 2014 | - |
| PEX19p | rabbit | 1:10000 | Colasante, 2017 |  |
| Complex IV | mouse | 1:500 | Invitrogen, 2015 | 459600 |
| SOD2 | rabbit | 1:500 | Abcam, 2016 | ab13533 |
| Vimentin | mouse | 1:5000 | Sigma, 2009 | v5255 |
| Pancytokeratin | mouse | 1:10000 | Abcam/Biozol 2006 | ab6401 |
| Amelogenin | mouse | 1:500 | Santa Cruz, 2019 | sc-365284 |
| Connexin 43 | rabbit | 1:400 | Sigma, 2011 | c6219 |
| Osteocalcin | goat | 1:300 | Acris, 2010 | BP712 |
| Osteopontin | rabbit | 1:200 | Acris, 2006 | R1565 |
| Ki-67 | rabbit | 1:100 | Abcam, 2019 | ab16667 |
| Caspase-3 | rabbit | 1:200 | Cell signalling, 2023 | #9661 |

**Supplementary Table S2:** secondary antibodies

| marking | fluorochrome | dilution | source and year of acquisition |
| --- | --- | --- | --- |
| donkey-anti-rabbit | Alexafluor 488 | 1:1000 | Molecular Probes, 2016 and 2018 |
| donkey-anti-mouse | Texas Red | 1:400 | Vector, 2016 |
| donkey-anti-goat | Alexafluor 594 | 1:300 | Molecular Probes 2013 |
| goat-anti-rat | Cy3 | 1:600 | Jackson/Dianova 2015 |
