# Supplementary figures and images for: Peroxisomal dysfunction interferes with odontogenesis and leads to developmentally delayed teeth and defects in distinct dental cells in Pex11b-deficient mice
Source: PLoS One. 2024 Dec 9;19(12):e0313445. doi: 10.1371/journal.pone.0313445 (PMC11627416; doi:10.1371/journal.pone.0313445)

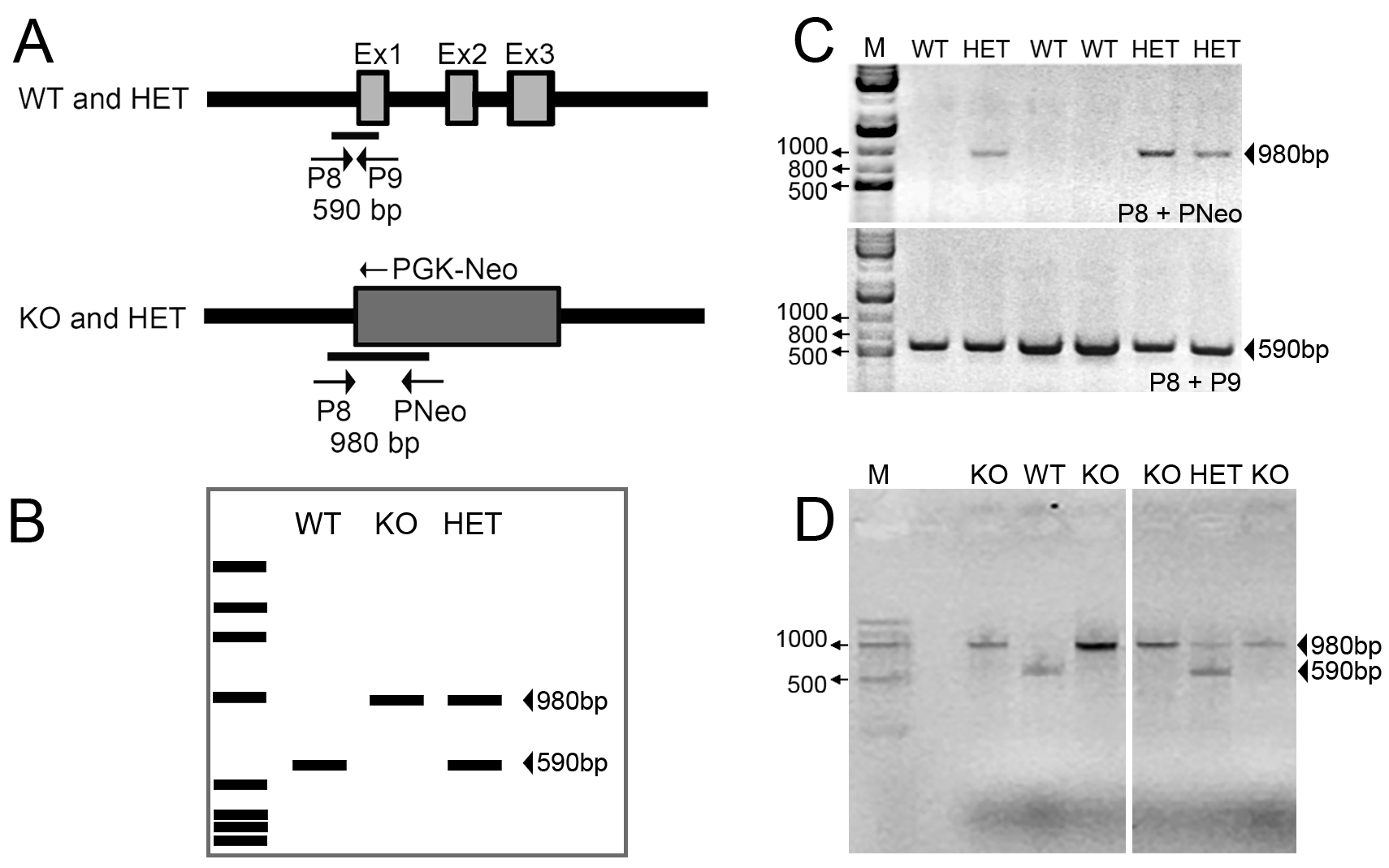

Supplement: S1 Fig — (A) Schematic representation of the PEX11b WT locus and KO locus (both present in the HET) showing the hybridization site of the primers P8, P9 and PNeo used for genotyping PCR. Size of the expected PCR fragments are indicated. (B) Schematic representation of the results expected when conducting a multiplex PCR on the PEX11b mice samples. For WT only the 590 bp band should appear, for HET both the 590 and the 980 bp bands and for the KO only the 980 bp band. (C) Individual PCR reactions for the genotyping of the WT and HET mice bred and embedded at the JLU just prior to this study. For these DNA samples PCR using either the primer combination P8 and P9 or P8 and PNeo were used to determine the presence of the WT and the KO gene locus. All probes show the expected bands. (D) Multiplex PCR of the DNA samples obtained from the PEX11b sections embedded in 2002. All probes show the expected bands. (TIF) [file pone.0313445.s002.tif]

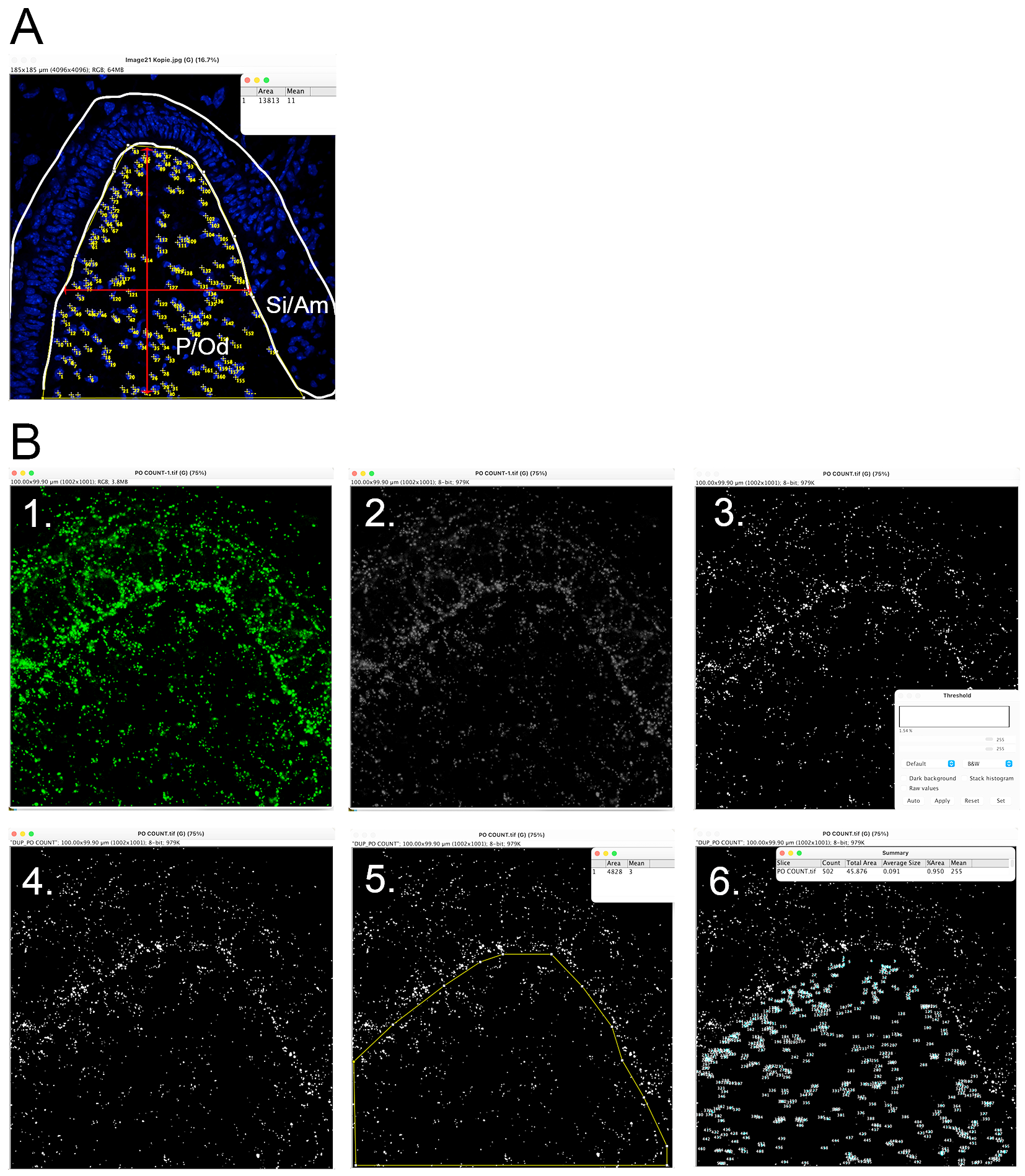

Supplement: S2 Fig — Representation of the steps involved during morphometric analysis of the number of nuclei and peroxisomes. (A): The number of the nuclei (nuclei/μm2) was determined using the “Multi-point” tool of image J for manual counting after selecting and measuring the ROI. (B): The number of peroxisomes was analyzed using the automatic “particle counting” function of Image J. Steps 1.-3.: To distinguish the organelles from the background, the images were converted into 8-bit images and then the threshold value was set. Step 4.: Individual peroxisomes that appeared to be fused were separated by converting the image to binary and applying watershed. Steps 5. And 6.: After setting the scale (μm) and selecting the ROI all particles with intensities above the threshold value and with a size range of 0.01–1 μm2 were automatically counted by image J. (TIF) [file pone.0313445.s003.tif]

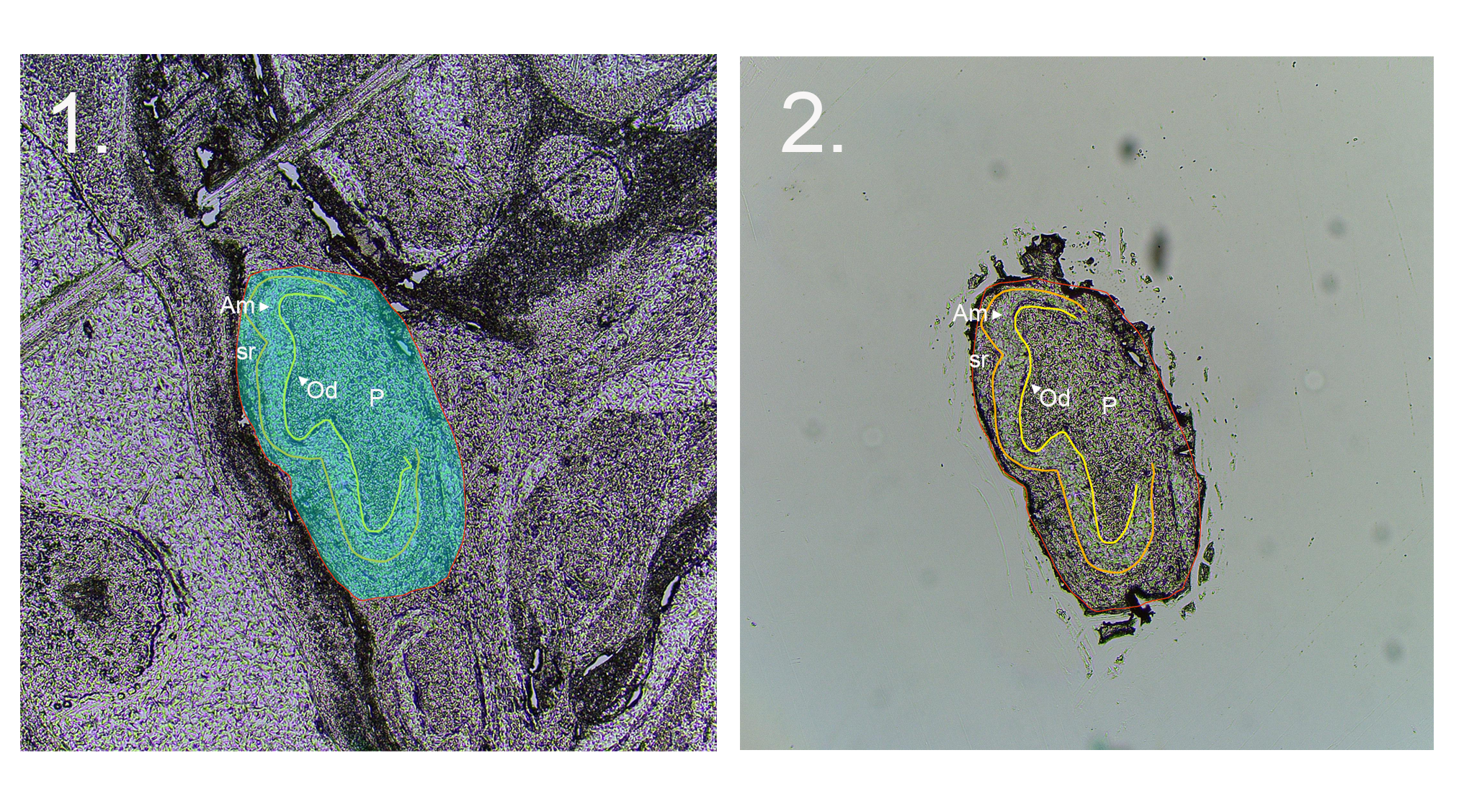

Supplement: S3 Fig — Exemplary image of microdissected tooth prior (Step 1.) and after (Step 2.) microdissection. The red outline filled with cyan background indicated the area of dissected tissue. The orange line indicates the stellate reticulum/ameloblast ameloblast border. The yellow line indicates the ameloblast/odontoblast border. Abbreviations: sr, stellate reticulum, Am, ameloblasts; Od, odontoblasts; P, dental pulp. (TIF) [file pone.0313445.s004.tif]

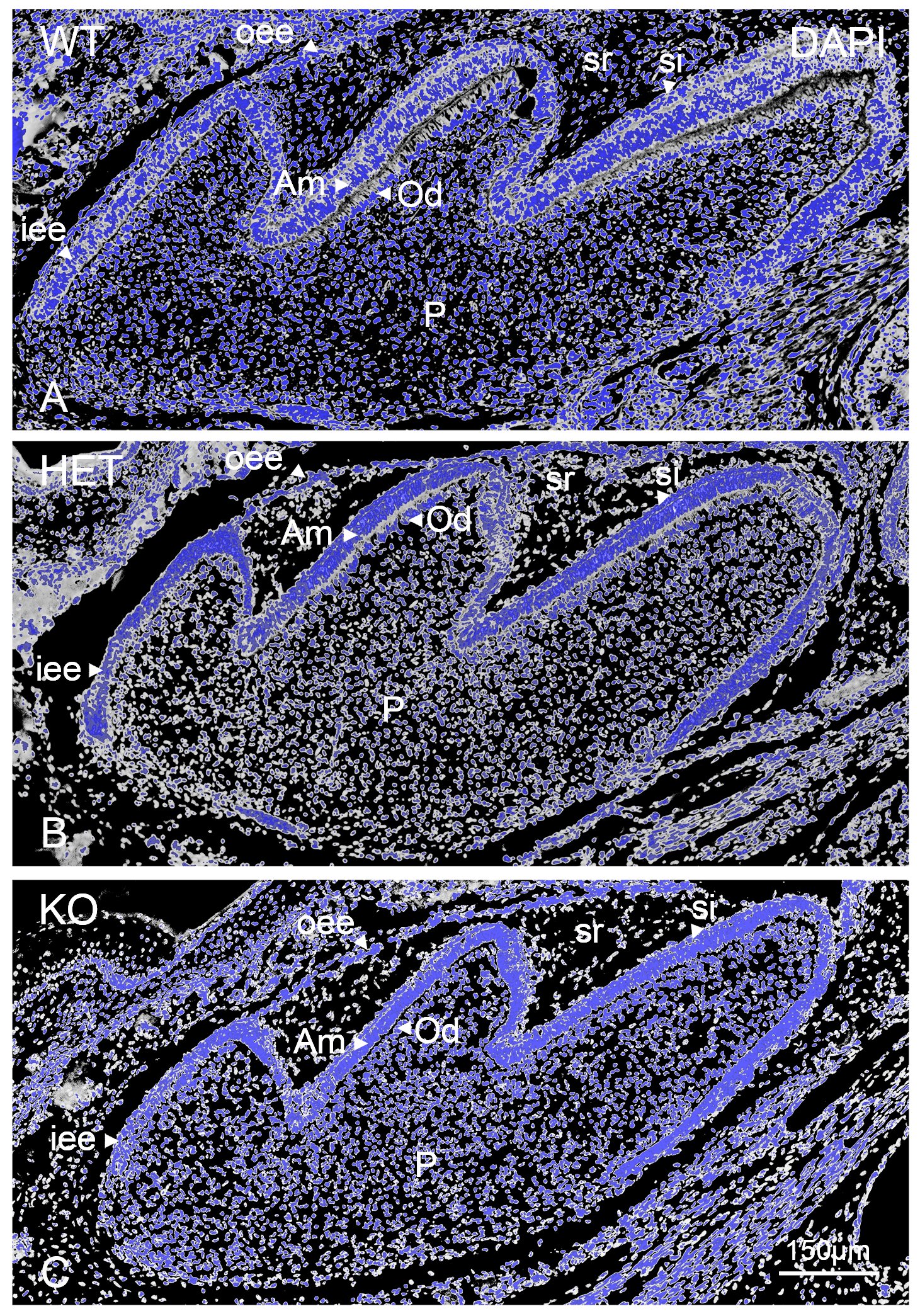

Supplement: S4 Fig — (A-C): DAPI-stainings of the first molars of wildtype (WT) (A), heterozygous (HET) (B) and knockout mice (KO) (C): The pictures show the typical cell types during the bell stage of odontogenesis: Outer enamel epithelium (oee, arrowheads), stellate reticulum (sr), stratum intermedium (si, arrowheads), inner enamel epithelium / ameloblasts (iee / Am, arrowheads), odontoblasts (Od, arrowheads) and dental pulp (P). The cytoplasmic background present in the images of Fig 3 was digitally amplified to highlight the areas of the individual cells (gray) and overlayed with the corresponding DAPI staining. (TIF) [file pone.0313445.s005.tif]

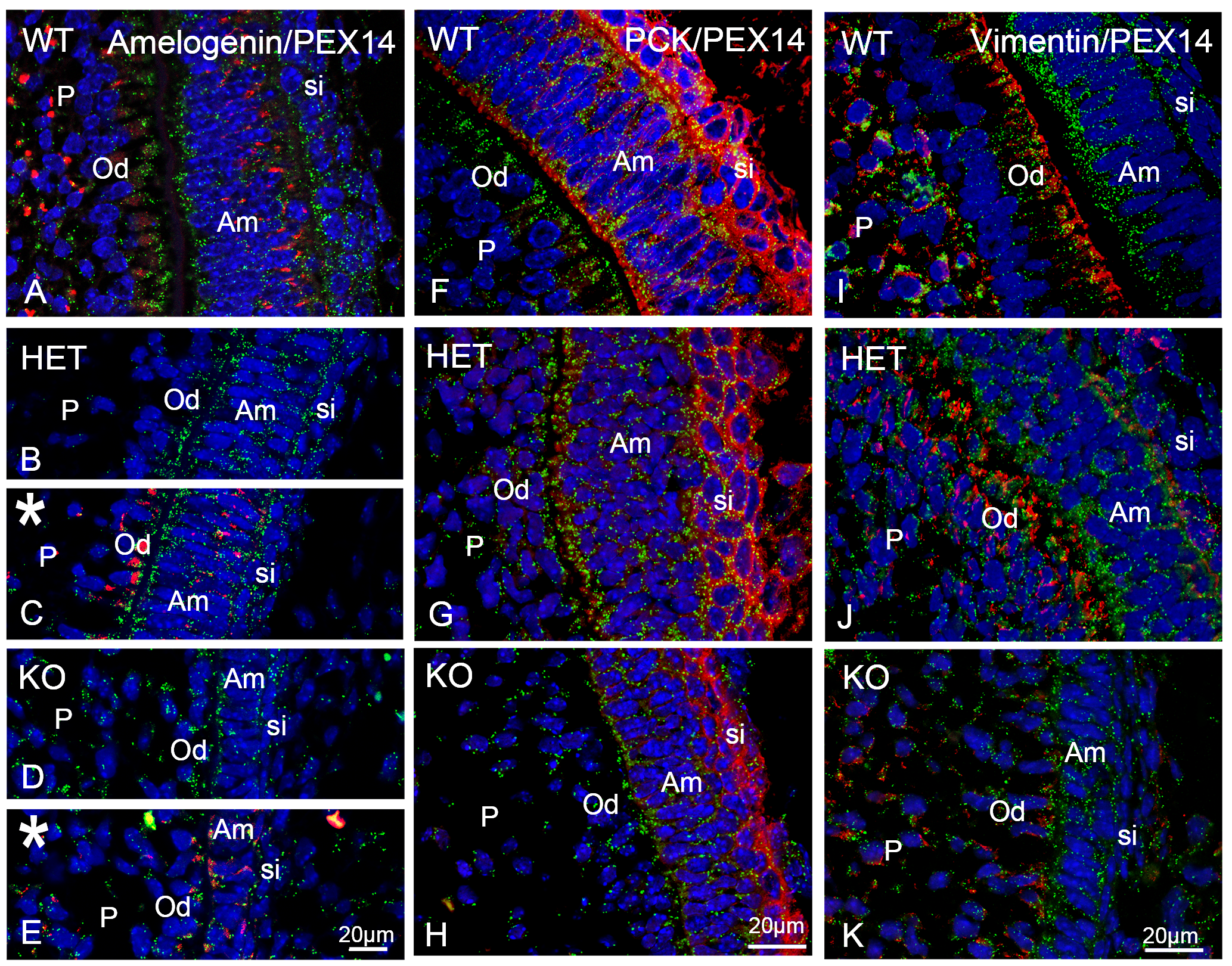

Supplement: S5 Fig — Immunofluorescence analyses of the peroxisomal marker PEX14b and amelogenin (A-E), pancytokeratin (F-H) and vimentin (I-K) in wildtype (WT), heterozygous (HET) and knockout (KO) mouse bell stage first molars. The pictures marked with an asterisk (*) represent the digitally intensified versions of (B) and (D). Bars shown in figures (E), (H) and (K) represent the magnification for the corresponding image columns of all three genotypes. DAPI was used to counterstain nuclei. Abbreviations: si, stratum intermedium, Am, ameloblasts; Od, odontoblasts; P, dental pulp. (TIF) [file pone.0313445.s006.tif]

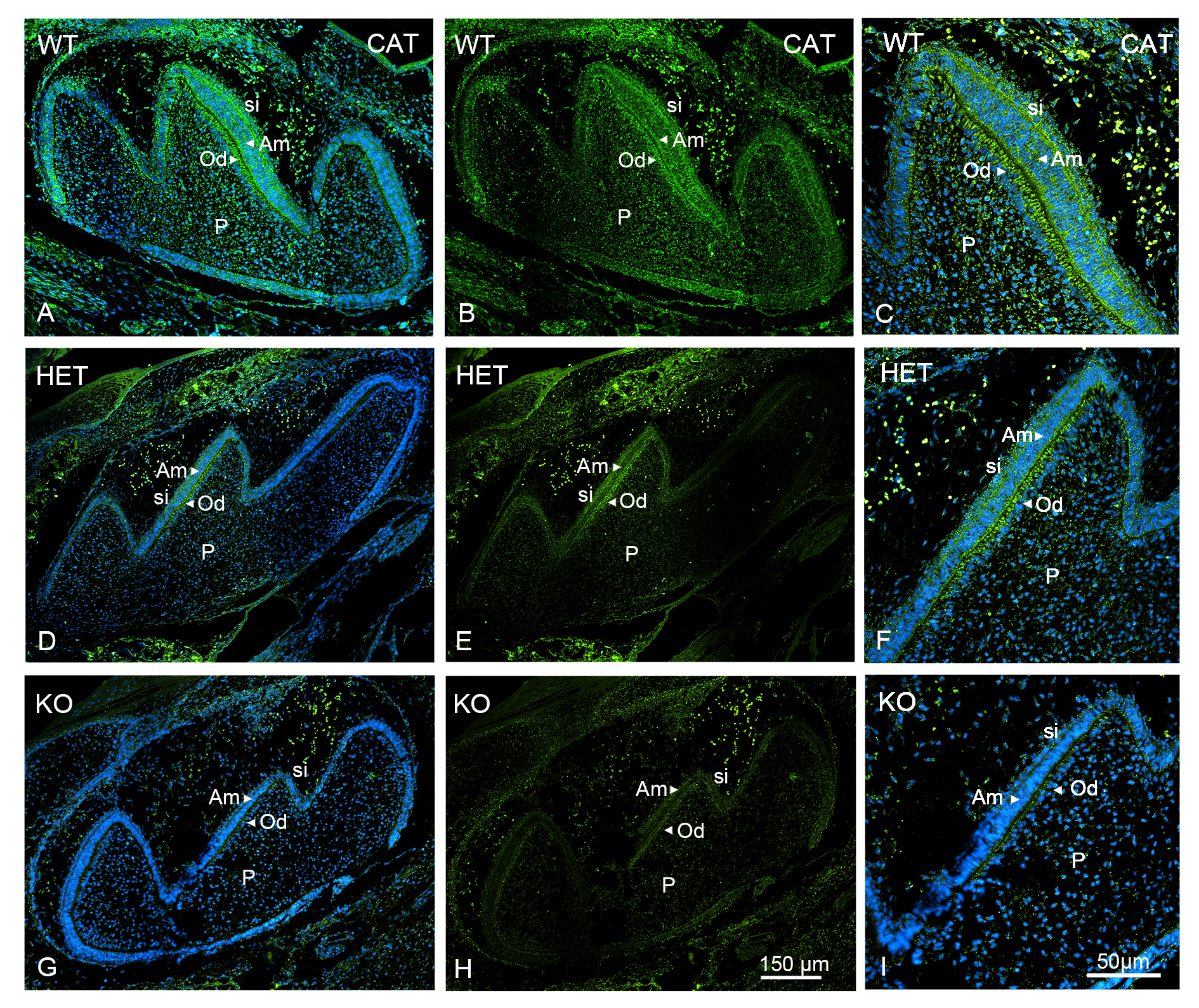

Supplement: S6 Fig — (A-I): Immunofluorescence analyses of catalase in wildtype (WT), heterozygous (HET) and knockout (KO) mouse bell stage first molars. Images B, E and H reflect catalase-stained images A, D and G respectively without the DAPI staining. Images C, F and I show higher magnification of the central dental cusp shown in A, D and G respectively. The bar shown in figure (H) indicates magnification for images A, B, D, E, G and H. The bar shown in Figure (I) represents the magnification the corresponding staining of all three genotypes. DAPI was used to counterstain nuclei. Abbreviations: si, stratum intermedium, Am, ameloblasts; Od, odontoblasts; P, dental pulp. (TIF) [file pone.0313445.s007.tif]

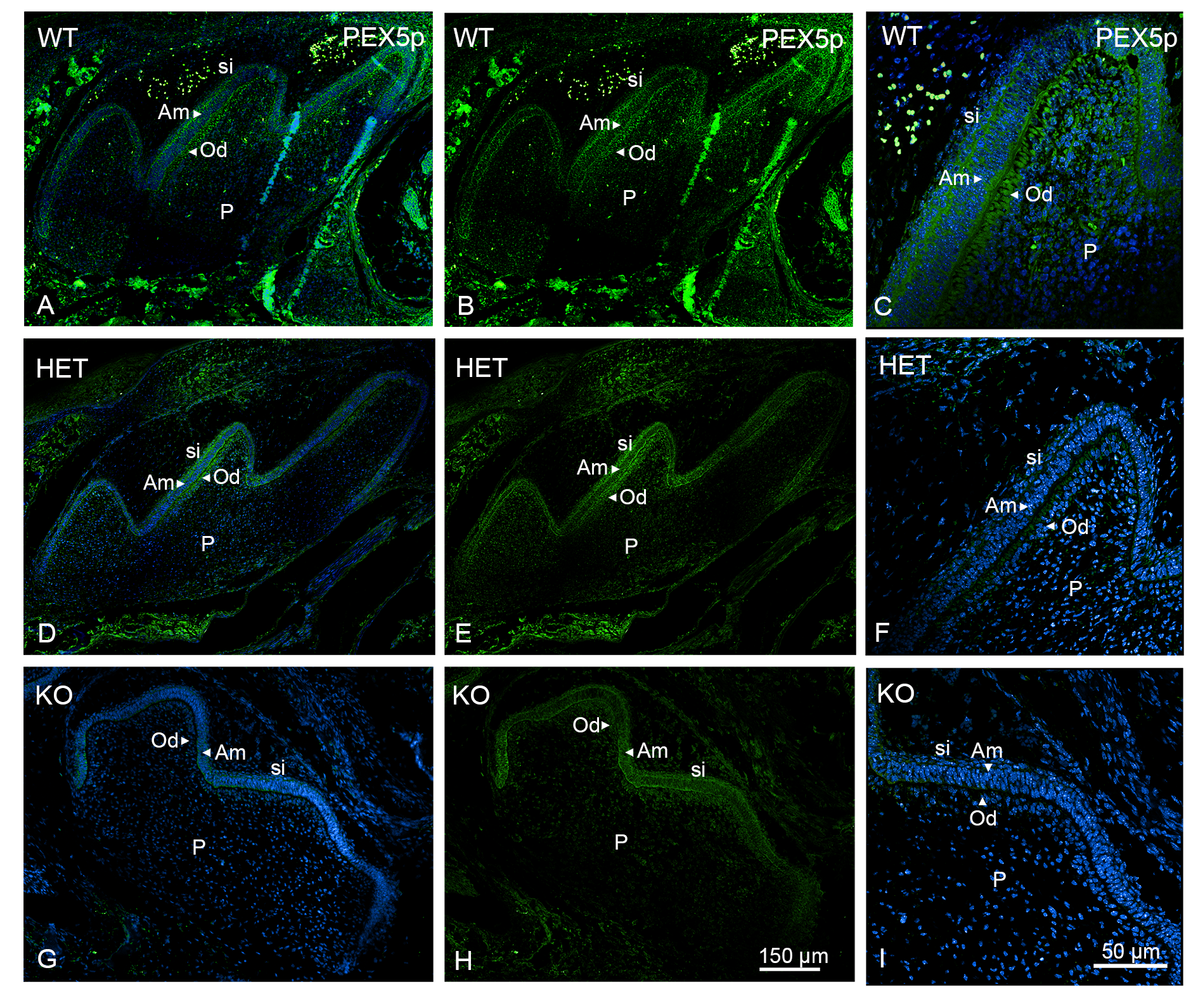

Supplement: S7 Fig — (A-I): Immunofluorescence analyses of PEX5 in wildtype (WT), heterozygous (HET) and knockout (KO) mouse bell stage first molars. Images B, E and H reflect PEX5-stained images A, D and G respectively without the DAPI staining. Images C, F and I show higher magnification of the central dental cusp shown in A, D and G respectively. The bar shown in figure (H) indicates magnification for images A, B, D, E, G and H. The bar shown in Figure (I) represents the magnification the corresponding staining of all three genotypes. DAPI was used to counterstain nuclei. Abbreviations: si, stratum intermedium, Am, ameloblasts; Od, odontoblasts; P, dental pulp. (TIF) [file pone.0313445.s008.tif]

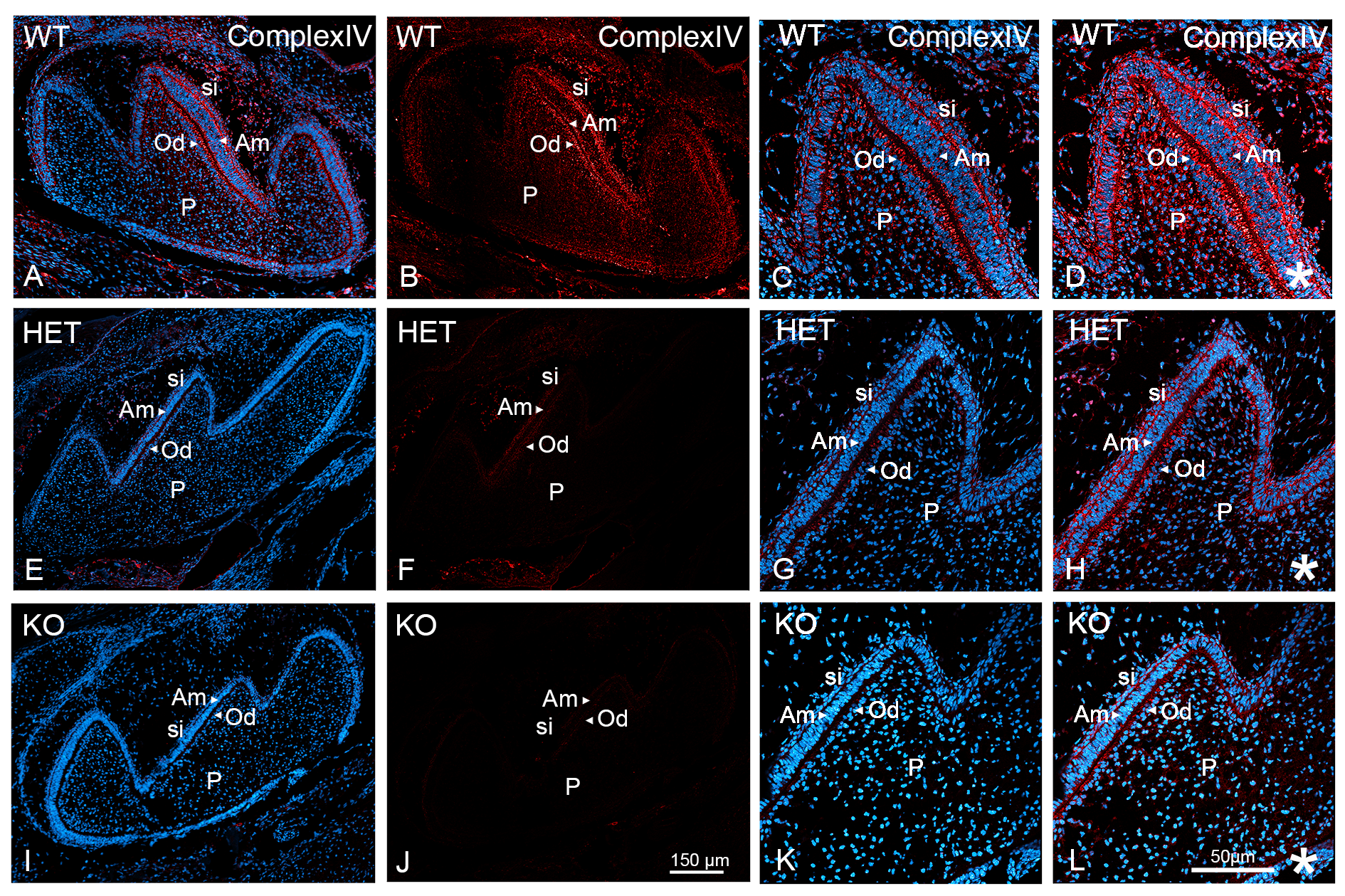

Supplement: S8 Fig — (A-L): Immunofluorescence analyses of complex IV in wildtype (WT), heterozygous (HET) and knockout (KO) mouse bell stage first molars. Images B, F and J reflect complex IV-stained images A, E and I respectively without the DAPI staining. Images C, G and K show higher magnification of the central dental cusp shown in A, E and I respectively. Images D, H and L show higher exposure of the central dental cusp shown in C, G and K respectively. The bar shown in figure (J) indicates magnification for images A, B, E, F, I and J. The bar shown in Figure (L) represents the magnification for the corresponding staining of C, D, G, H, K and L. DAPI was used to counterstain nuclei. Abbreviations: si, stratum intermedium, Am, ameloblasts; Od, odontoblasts; P, dental pulp; * higher exposure. (TIF) [file pone.0313445.s009.tif]

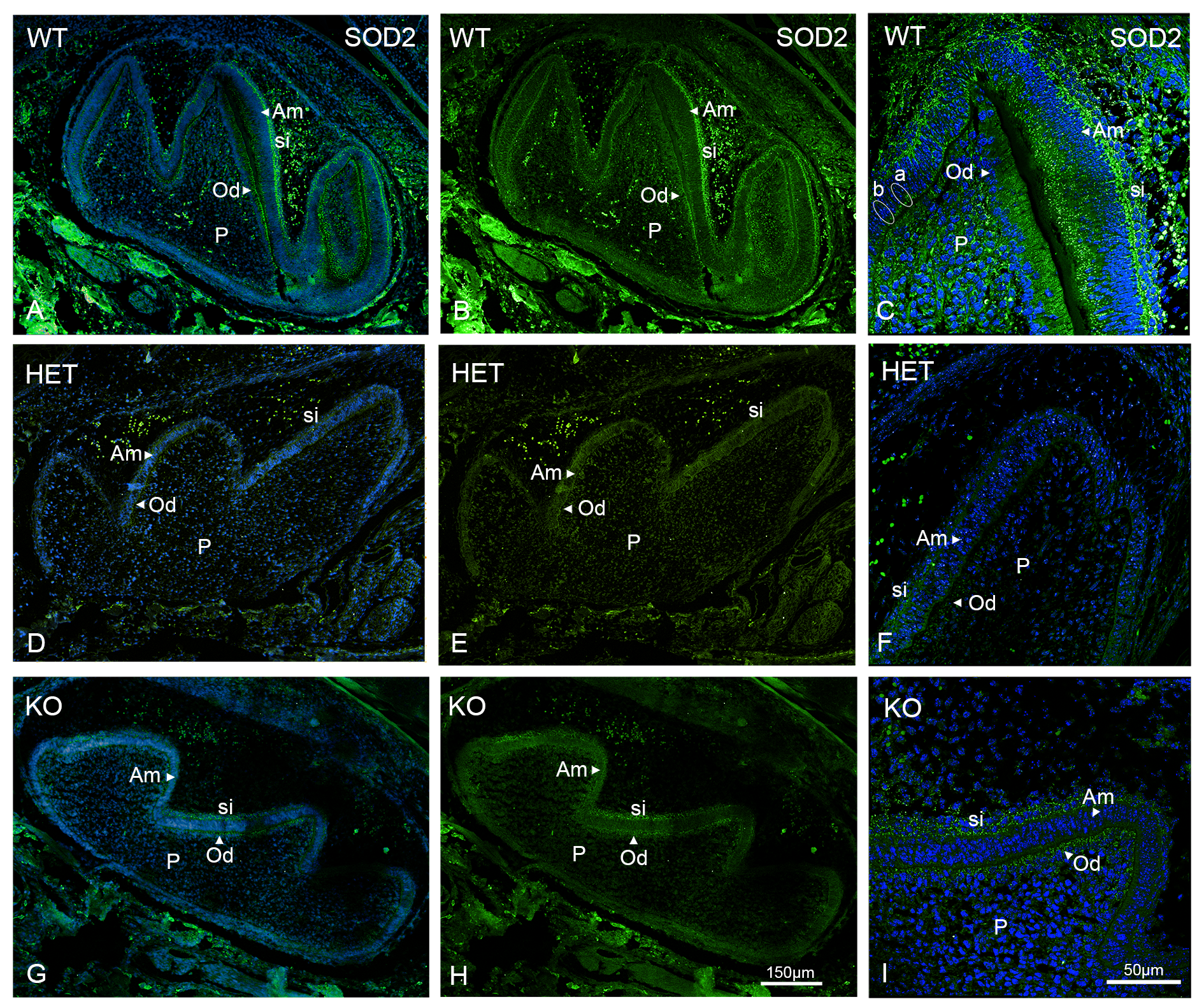

Supplement: S9 Fig — (A-I): Immunofluorescence analyses of SOD2 in wildtype (WT), heterozygous (HET) and knockout (KO) mouse bell stage first molars. Images B, E and H reflect SOD2-stained images A, D and G respectively without the DAPI staining. Images C, F and I show higher magnification of one of the dental cusps shown in A, D and G respectively. The bar shown in figure (H) indicates magnification for images A, B, D, E, G and H. The bar shown in Figure (I) represents the magnification for the corresponding staining of all three genotypes. DAPI was used to counterstain nuclei. Abbreviations: si, stratum intermedium, Am, ameloblasts; Od, odontoblasts; P, dental pulp. a, ameloblast with high number of SOD2-stained mitochondria; b, ameloblast with low number of SOD2-stained mitochondria. (TIF) [file pone.0313445.s010.tif]

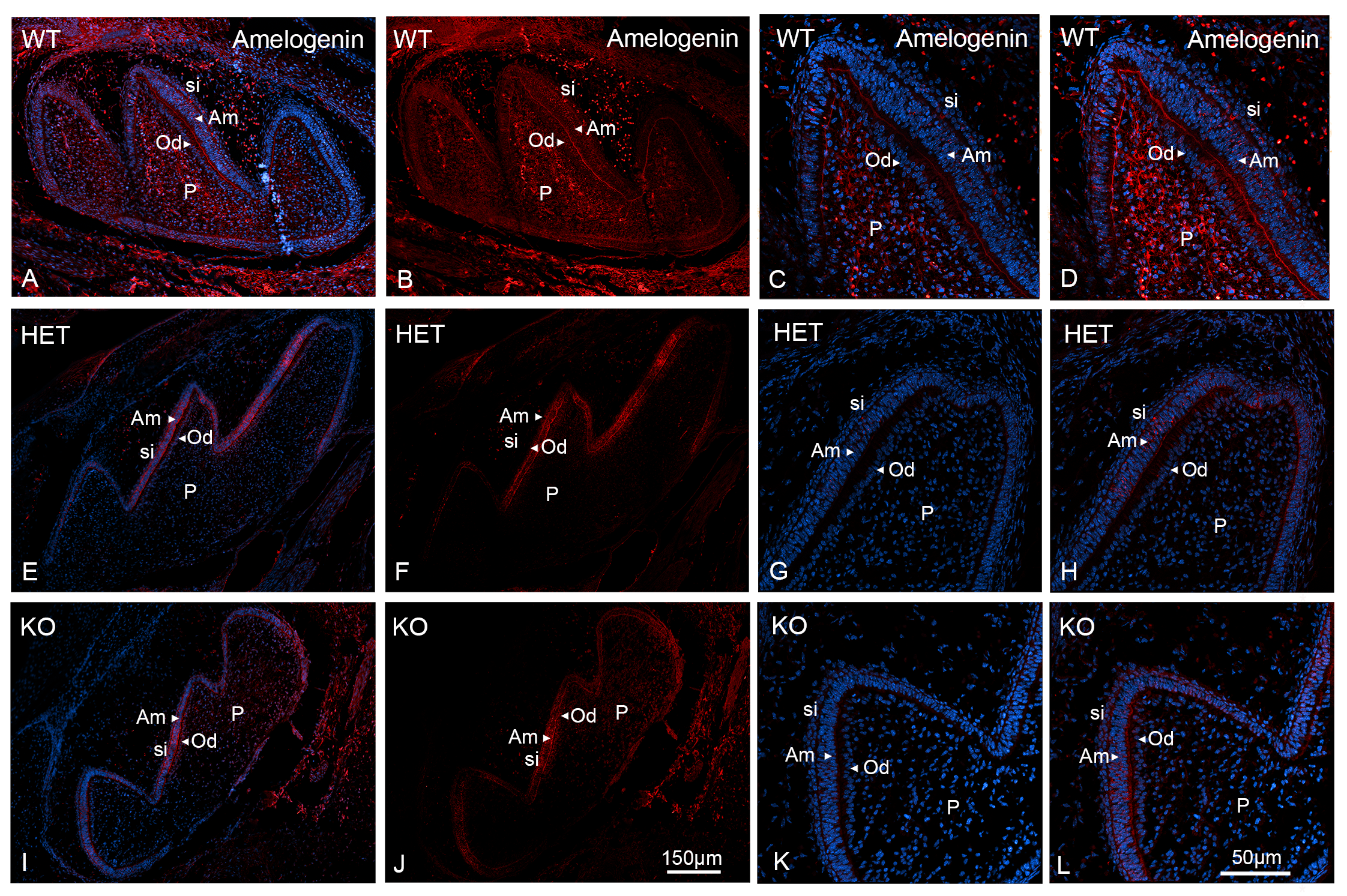

Supplement: S10 Fig — (A-L): Immunofluorescence analyses of amelogenin in wildtype (WT), heterozygous (HET) and knockout (KO) mouse bell stage first molars. Images B, F and J reflect amelogenin-stained images A, E and I respectively without the DAPI staining. Images C, G and K show higher magnification of one of the dental cusps shown in A, E and I respectively. Images D, H and L show higher exposure of the dental cusp shown in C, G and K respectively. The bar shown in figure (J) indicates magnification for images A, B, E, F, I and J. The bar shown in Figure (L) represents the magnification for the corresponding staining of C, D, G, H, K and L. DAPI was used to counterstain nuclei. Abbreviations: si, stratum intermedium, Am, ameloblasts; Od, odontoblasts; P, dental pulp; * higher exposure. (TIF) [file pone.0313445.s011.tif]

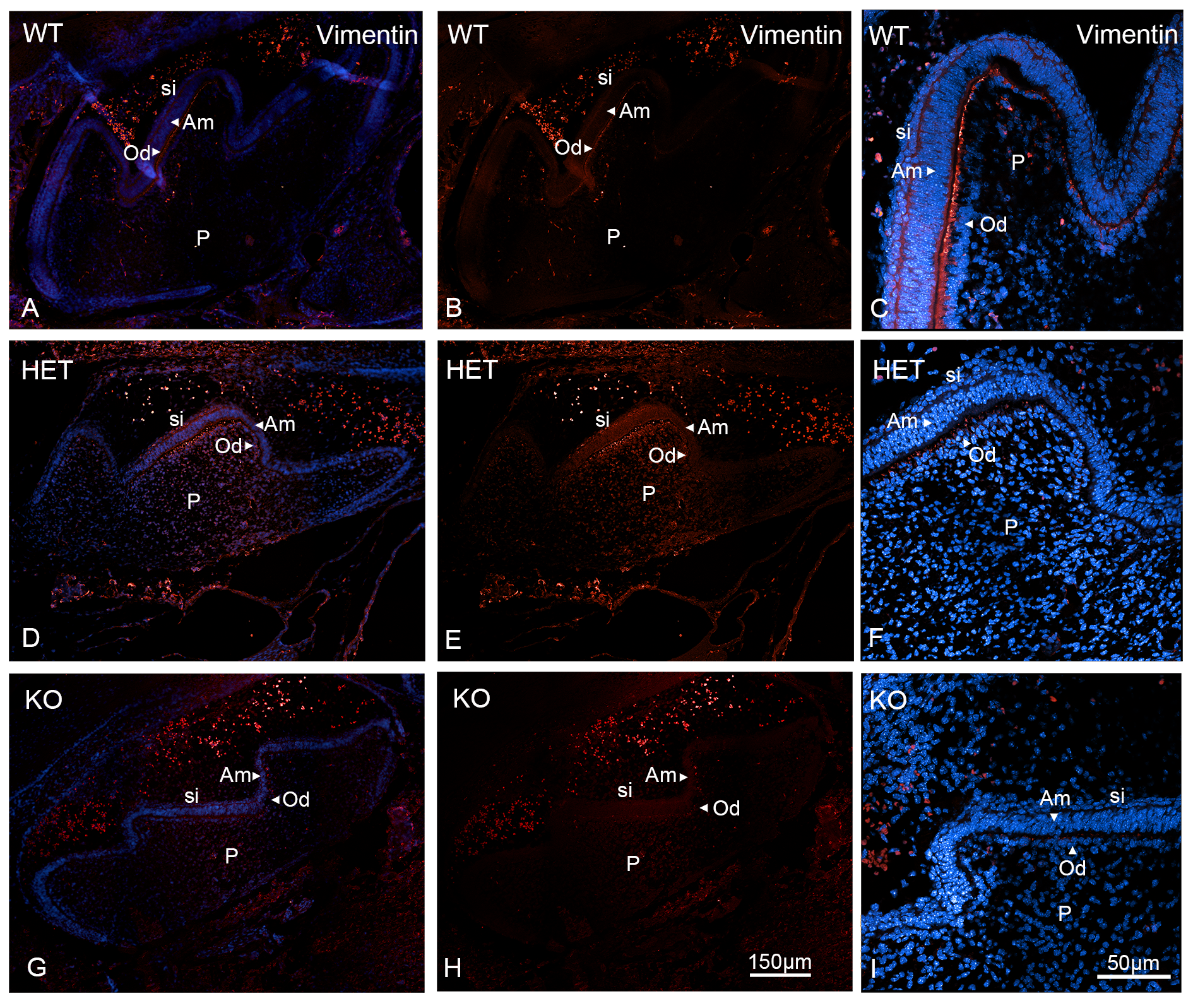

Supplement: S11 Fig — (A-I): Immunofluorescence analyses of vimentin in wildtype (WT), heterozygous (HET) and knockout (KO) mouse bell stage first molars. Images B, E and H reflect vimentin-stained images A, D and G respectively without the DAPI staining. Images C, F and I show higher magnification of the central dental cusp shown in A, D and G respectively. The bar shown in figure (H) indicates magnification for images A, B, D, E, G and H. The bar shown in Figure (I) represents the magnification for the corresponding staining of all three genotypes. DAPI was used to counterstain nuclei. Abbreviations: si, stratum intermedium, Am, ameloblasts; Od, odontoblasts; P, dental pulp. (TIF) [file pone.0313445.s012.tif]

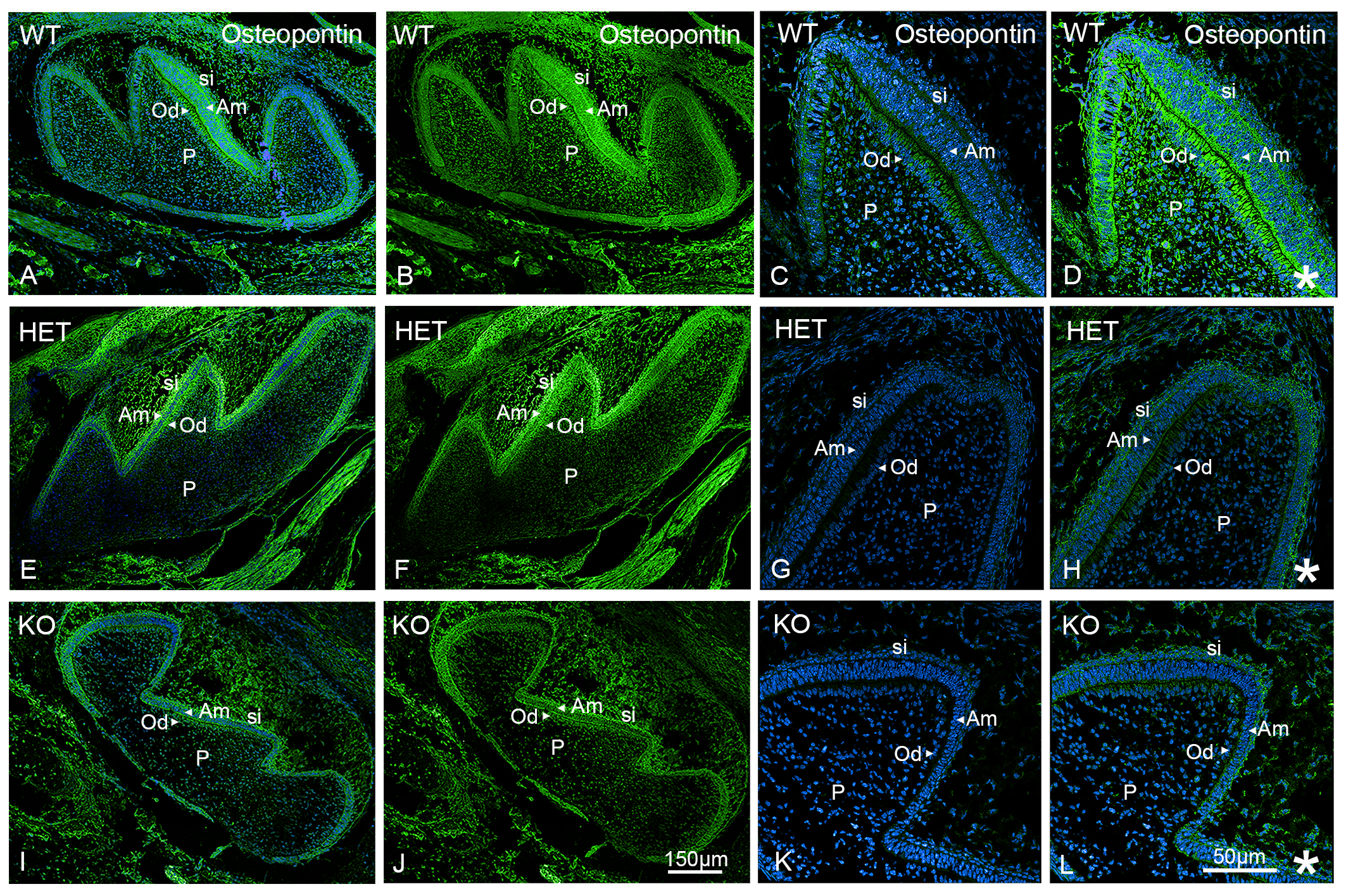

Supplement: S12 Fig — (A-L): Immunofluorescence analyses of osteopontin in wildtype (WT), heterozygous (HET) and knockout (KO) mouse bell stage first molars. Images B, F and J reflect osteopontin-stained images A, E and I respectively without the DAPI staining. Images C, G and K show higher magnification of one of the dentals cusp shown in A, E and I respectively. Images D, H and L show higher exposure of the dental cusps shown in C, G and K respectively. The bar shown in figure (J) indicates magnification for images A, B, E, F, I and J. The bar shown in Figure (L) represents the magnification for the corresponding staining of C, D, G, H, K and L. DAPI was used to counterstain nuclei. Abbreviations: si, stratum intermedium, Am, ameloblasts; Od, odontoblasts; P, dental pulp; * higher exposure. (TIF) [file pone.0313445.s013.tif]

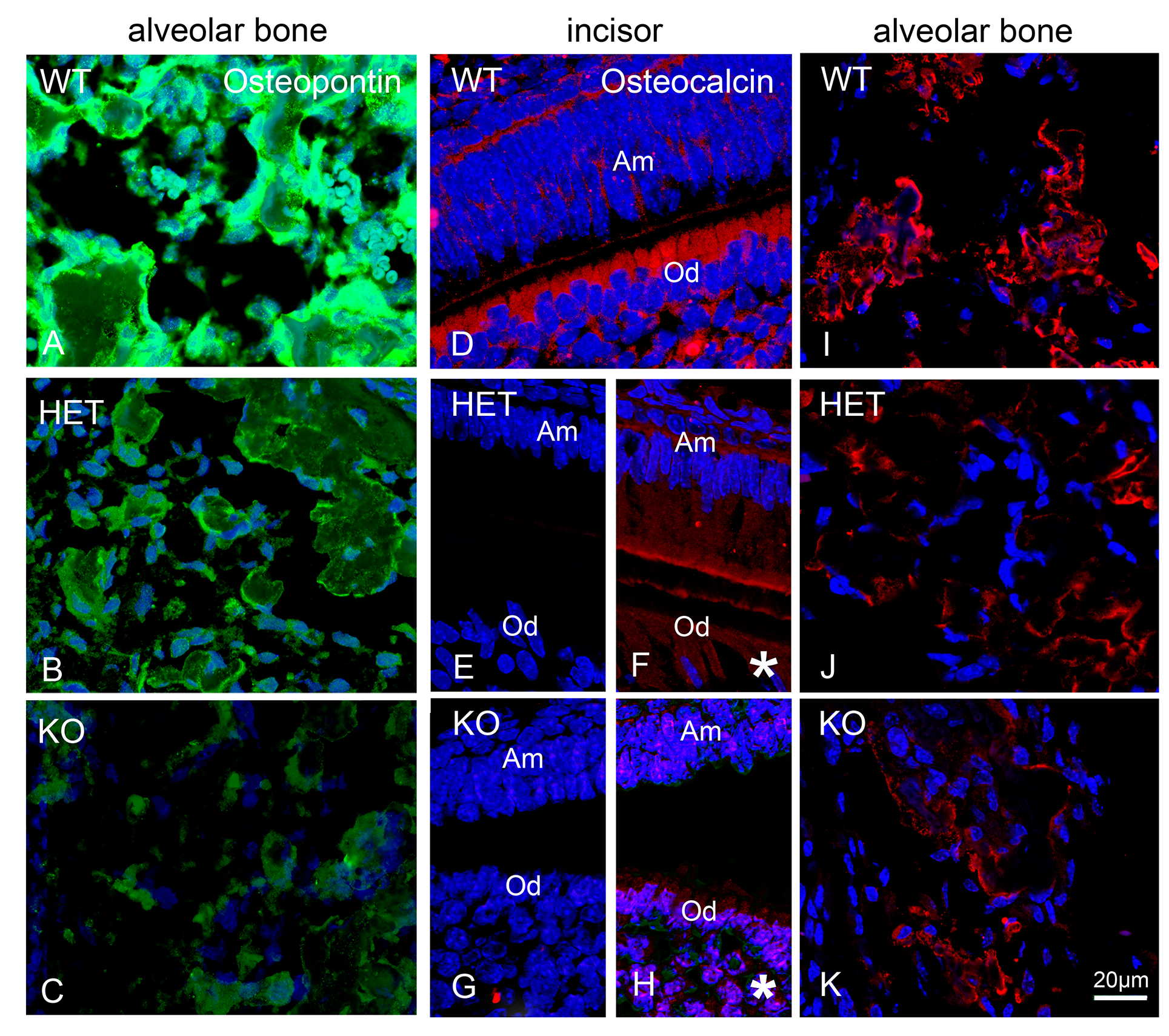

Supplement: S13 Fig — (A-C): Immunofluorescence analyses of osteopontin in alveolar bone in wildtype (WT), heterozygous (HET) and knockout (KO) mice alveolar bone. (D-K): Immunofluorescence analysis of osteocalcin in wildtype (WT), heterozygous (HET) and knockout (KO) mice bell stage incisors (D-H) and alveolar bone (I-K). Bar shown in figure K represents the magnification for all images. DAPI was used to counterstain nuclei. The pictures marked with an asterisk (*) represent higher exposures of the images shown in “E” and “G”. Abbreviations: Am, ameloblasts; Od, odontoblasts. (TIF) [file pone.0313445.s014.tif]

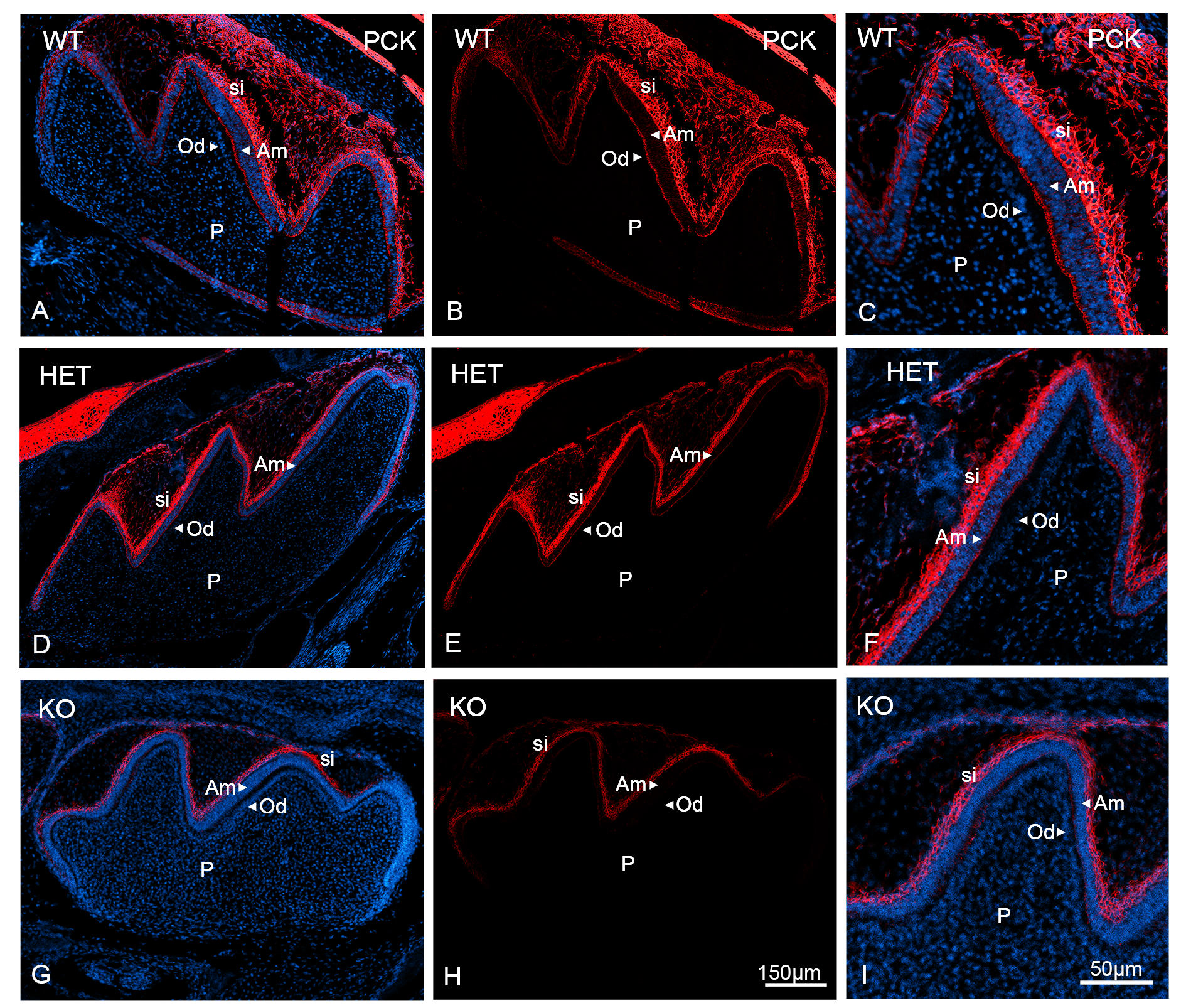

Supplement: S14 Fig — (A-I): Immunofluorescence analyses of pancytokeratin in wildtype (WT), heterozygous (HET) and knockout (KO) mouse bell stage first molars. Images B, E and H reflect pancytokeratin-stained images A, D and G respectively without the DAPI staining. Images C, F and I show higher magnification of one of the dental cusps shown in A, D and G respectively. The bar shown in figure (H) indicates magnification for images A, B, D, E, G and H. The bar shown in Figure (I) represents the magnification for the corresponding staining of all three genotypes. DAPI was used to counterstain nuclei. Abbreviations: si, stratum intermedium, Am, ameloblasts; Od, odontoblasts; P, dental pulp. (TIF) [file pone.0313445.s015.tif]

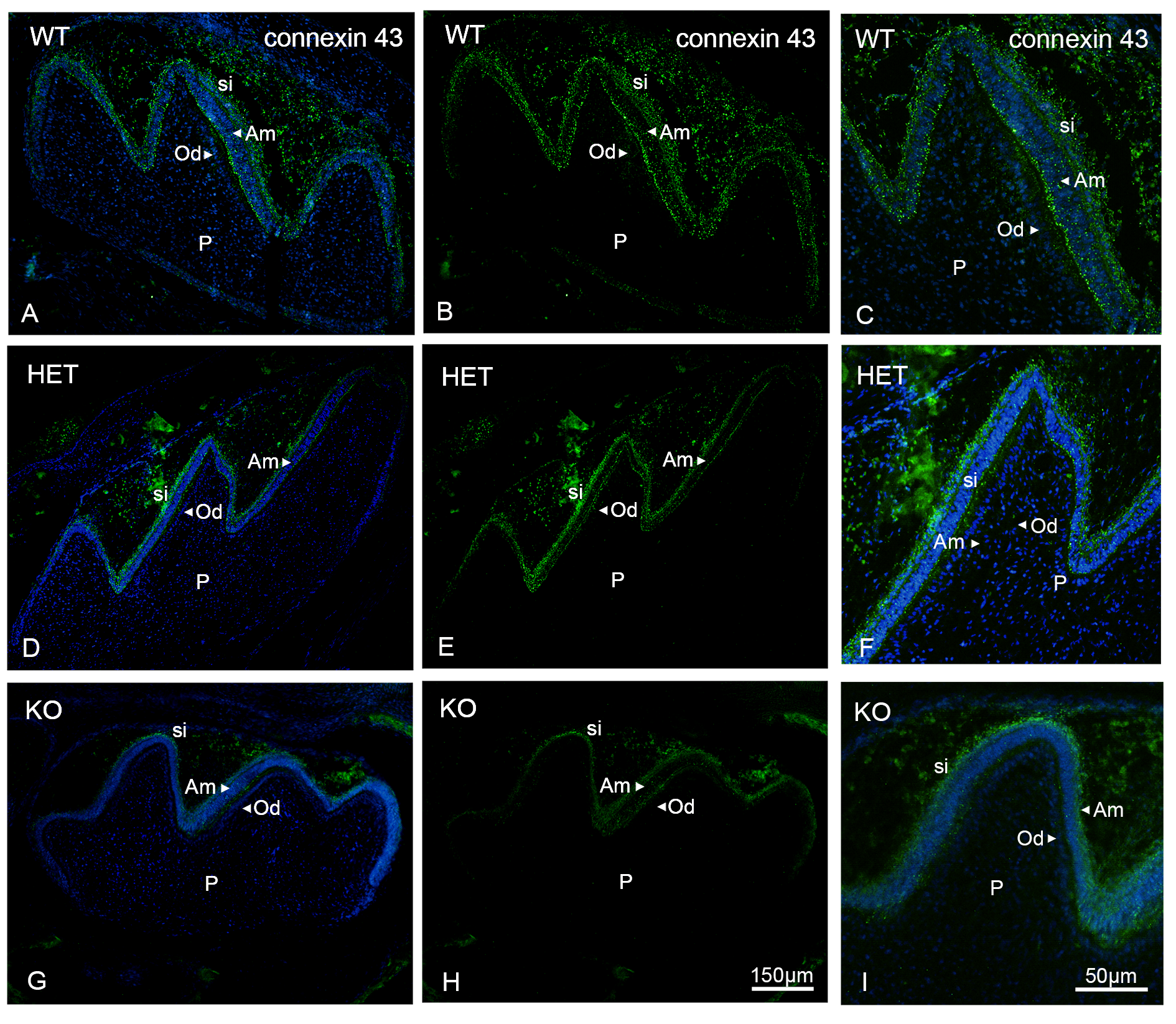

Supplement: S15 Fig — (A-I): Immunofluorescence analyses of connexin 43 in wildtype (WT), heterozygous (HET) and knockout (KO) mouse bell stage first molars. Images B, E and H reflect connexin 43-stained images A, D and G respectively without the DAPI staining. Images C, F and I show higher magnification of one of the dental cusps shown in A, D and G respectively. The bar shown in figure (H) indicates magnification for images A, B, D, E, G and H. The bar shown in Figure (I) represents the magnification for the corresponding staining of all three genotypes. DAPI was used to counterstain nuclei. Abbreviations: si, stratum intermedium, Am, ameloblasts; Od, odontoblasts; P, dental pulp. (TIF) [file pone.0313445.s016.tif]
